# Visible Light-Driven Reductive Azaarylation of Coumarin-3-carboxylic Acids

**DOI:** 10.1021/acs.joc.2c00683

**Published:** 2022-07-12

**Authors:** Ewelina Kowalska, Angelika Artelska, Anna Albrecht

**Affiliations:** † Institute of Organic Chemistry, Faculty of Chemistry, Lodz University of Technology, Żeromskiego 116, Łódź 90-924, Poland; ‡ Institute of Applied Radiation Chemistry, Lodz University of Technology, Żeromskiego 116, Łódź 90-924, Poland; § Institute of General and Ecological Chemistry, Faculty of Chemistry, Lodz University of Technology, Żeromskiego 116, Łódź 90-924, Poland

## Abstract

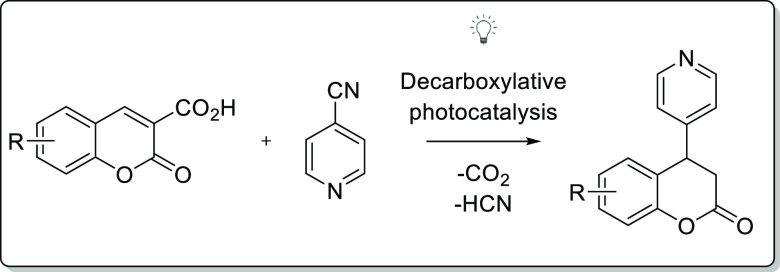

In the manuscript, reductive and decarboxylative azaarylation of coumarin-3-carboxylic acids is described. It utilizes the photocatalytic activation of (cyano)azaarenes in the presence of *fac*-Ir(ppy)_3_ as a photocatalyst. The methodology is versatile and provides access to biologically relevant 4-substituted-chroman-2-ones. Visible light, photoredox catalyst, base, anhydrous solvent, and inert atmosphere constitute key parameters for the success of the described strategy. The developed methodology involves a wide range of coumarin-3-carboxylic acids as well as (cyano)azaarenes.

## Introduction

**Scheme 1:**
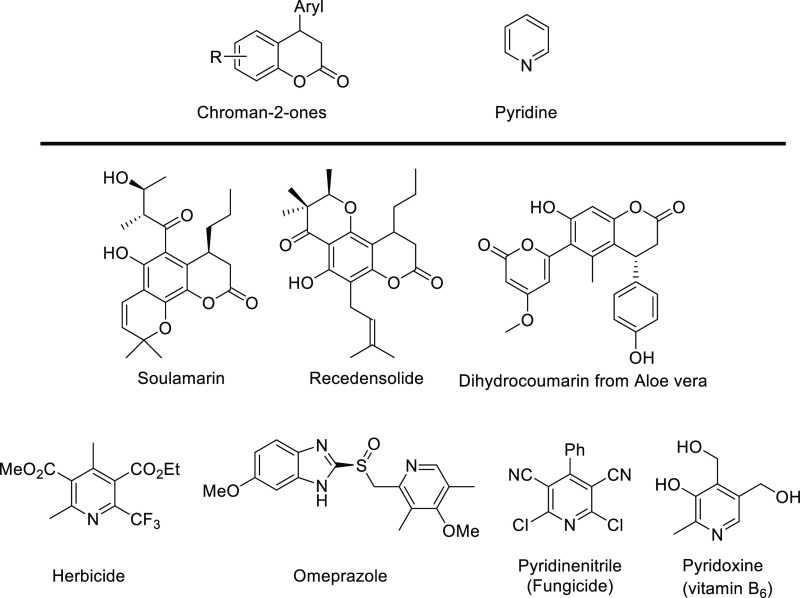
Importance of Chroman-2-one and Pyridine Derivatives

Chroman-2-one, pyridine, and their derivatives constitute privileged structural motifs present in various natural products.^1^ Representative examples of both groups of compounds are shown in Scheme 1. Although these compounds are abundant in nature, synthetic methods for their preparation are of importance.^2^ In this context, it is worth noting that pyridine is the second most frequent nitrogen-containing heterocyclic scaffold that is present in 62 U.S. FDA approved drugs displaying a wide range of biological activities.^3^ Thus, the pyridine skeleton often serves as a “privileged” scaffold in drug design and discovery. Moreover, it is also a versatile building block utilized for the synthesis of chiral ligands applied in asymmetric catalysis.^4^ As a consequence, a lot of effort has been devoted toward the development of methods for the synthesis of pyridine derivatives.^5^ Recently, radical-based pyridylation reactions have attracted much attention, providing a flexible approach to pyridine derivatives by the application of photocatalysis. These strategies benefit from good functional group tolerance, procedural simplicity, and mild reaction conditions.^6^

**Scheme 2:**
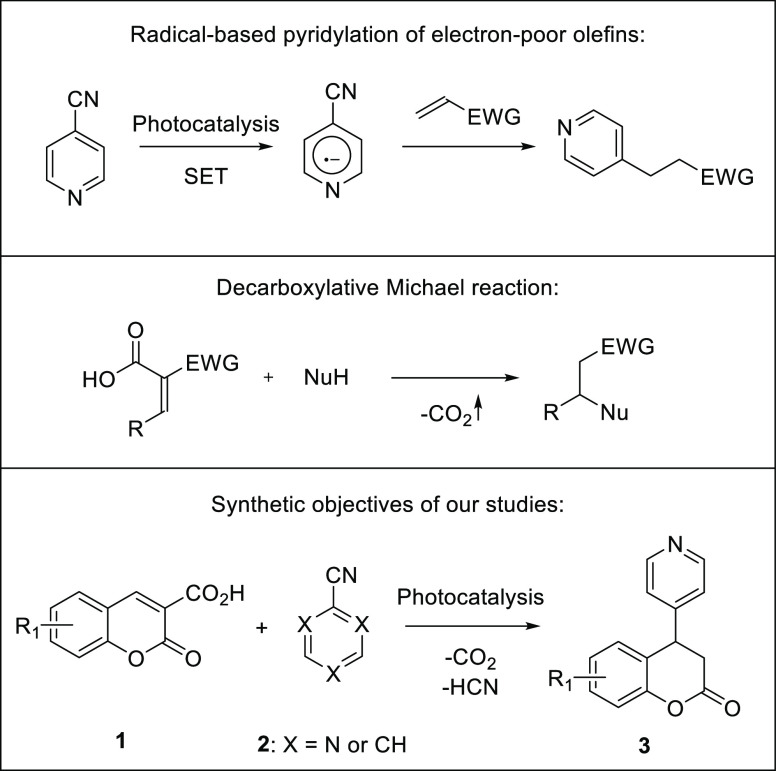
Importance of Decarboxylative Approaches in Organic Synthesis and the Objectives of Our Study

The addition of free radicals to electron-deficient olefins is known as Giese reaction (Scheme 2).^7^ Recent advancements in this field arise from the development of photo-mediated methods allowing for the free-radical formation under mild and nontoxic conditions.^8^

A decarboxylative Michael reaction based on nucleophilic addition to carboxylic-acid-activated olefins followed by a decarboxylation reaction constitutes a powerful synthetic tool.^9^ Recently, we described the first photocatalytic, doubly decarboxylative Giese reaction applicable to a wide range of carboxylic acids.^10^ Coumarin-3-carboxylic acids **1** constitute useful acceptors in this reaction, opening access to biologically relevant chroman-2-ones **3**.^11^ Given the interesting properties of coumarin and pyridine derivatives, the task of development of synthetic routes leading to hybrid molecules bearing both structural motifs was undertaken. Notably, the synthesis of hybrid molecules containing more than one biologically active unit constitutes an important approach in modern drug design.^12^

Herein, we present our studies on the development of decarboxylative reductive arylation of coumarin-3-carboxylic acids. (Cyano)azaarenes were applied as nucleophiles in the Giese-type transformation. This methodology benefits from mild reaction conditions and a broad scope of substrates.

## Results and Discussion

Initially, reactions between cyanopyridine **2a** and coumarin derivatives **1** bearing either no or various activating groups in the 3-position were performed (Table 1, entries 1–4). Experiments were performed in acetonitrile in the presence of **4a** as a photocatalyst and triethylamine as a base under irradiation with blue light and an inert atmosphere at room temperature. When simple coumarin **5a** was used, no reaction was observed. Therefore, EWG-activated coumarin derivatives **1b**–**e** were tested. Surprisingly, derivatives **5b**–**d** displayed no reactivity under these conditions. To our delight, the incorporation of the carboxylic acid moiety into the structure of coumarin **1a** resulted in the formation of the desired product **3aa**, indicating the crucial role of the carboxylic-acid-group activation in the devised reactivity (Table 1, entry 5). Further optimization studies were performed using coumarin-3-carboxylic acid **1a** and 4-cyanopyridine **2a** as model substrates (Table 1, entries 6–22). In the first part of the optimization studies, the catalytic activity of six different photoredox catalysts was tested (with the irradiation with the light source of suitable wavelength) (Table 1, entries 5–10). When Eosin Y **4b** was used, the formation of target product **3aa** was not observed (Table 1, entry 6). Catalysts **4a** and **4c**–**f** provided the desired reactivity (Table 1, entries 5 and 7–10, respectively) with the best results obtained in the presence of catalysts **4a** (Table 1, entry 5). In the course of further studies, the amount of 4-cyanopyridine **2a** was tested. It was shown that the reaction with a 3-fold excess of **2a** gave the product **3aa** with 49% yield (Table 1, entry 11). Further increasing the amount of 4-cyanopyridine **2a** did not improve the result. In the next step of optimization studies, the effect of the solvent on the reaction outcome was evaluated (Table 1, entries 11–16). The use of different solvents ensured the product formation; however, the best result was obtained when dimethyl sulfoxide was employed (Table 1, entry 13). During further investigations, the amount of catalyst **4a** was studied (Table 1, entries 16–18). It proved possible to be lowered to 3 mol % without a significant change of the result (Table 1, entry 17). Furthermore, the effect of base on the reaction outcome was evaluated (Table 1, entries 19–21). When DABCO was used, product **3aa** was not formed (Table 1, entry 20) and the application of DIPEA and *N*-methyl morpholine resulted in diminished yields (Table 1, entries 19 and 21). In the course of further studies, control experiments were performed (Table 1, entries 24–26). The use of stoichiometric amount of Et_3_N yielded the product **3aa** with low yield (25%) (Table 1, entry 24). The reaction did not proceed in the absence of photoredox catalysts (Table 1, entry 25). A similar effect was observed when the transformation was attempted in the dark (Table 1, entry 26), thus confirming the crucial effect of photocatalyst and the source of light on the reaction outcome. Notably, the optimized reaction proved readily scalable to a 2 mmol scale and the product **3aa** was obtained with a high yield (Table 1, entry 27). Finally, the experiment in the presence of TEMPO was carried out and no reaction was observed, thus confirming the radical nature of the developed reaction (Table 1, entry 28).

**Table 1:**
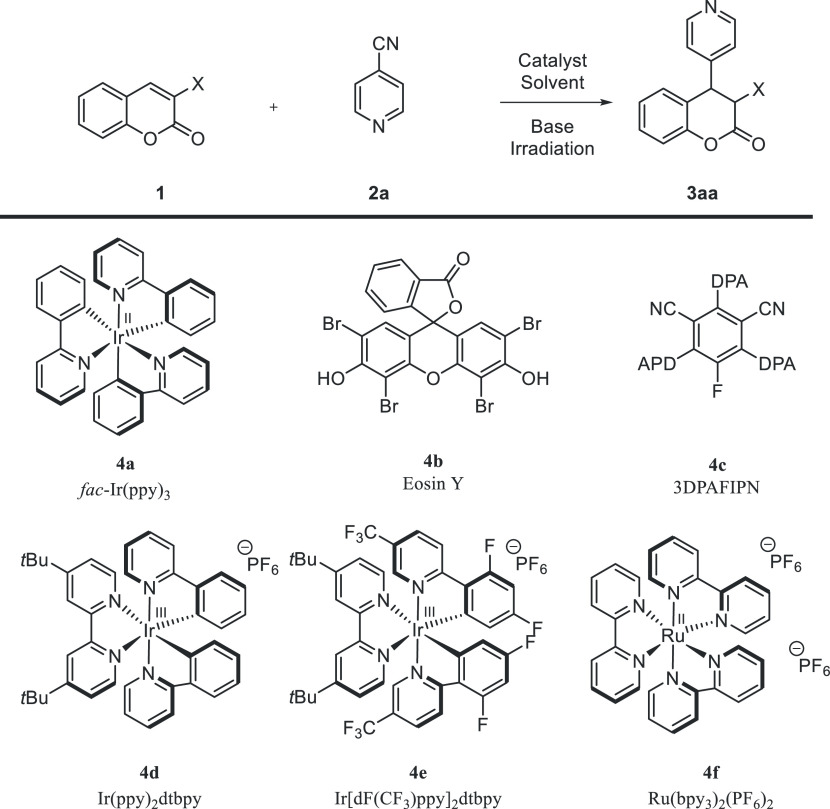
Visible Light-Driven Reductive Azaarylation of Coumarin **1a** and Its Derivatives **5a**–**d**: Optimization studies^a^

entry	catalyst	X	solvent	base	catalyst [mol %]	yield [%]
1^b^	**4a**	H (**5a**)	CH_3_CN	Et_3_N	10	
2^b^	**4a**	CN (**5b**)	CH_3_CN	Et_3_N	10	
3^b^	**4a**	CO_2_Et (**5c**)	CH_3_CN	Et_3_N	10	
4^b^	**4a**	C(O)Ph (**5d**)	CH_3_CN	Et_3_N	10	
5^b^	**4a**	CO_2_H (**1a**)	CH_3_CN	Et_3_N	10	30
6^c^	**4b**	CO_2_H (**1a**)	CH_3_CN	Et_3_N	10	
7^b^	**4c**	CO_2_H (**1a**)	CH_3_CN	Et_3_N	10	21
8^b^	**4d**	CO_2_H (**1a**)	CH_3_CN	Et_3_N	10	14
9^b^	**4e**	CO_2_H (**1a**)	CH_3_CN	Et_3_N	10	24
10^b^	**4f**	CO_2_H (**1a**)	CH_3_CN	Et_3_N	10	12
11^b^^,^^d^	**4a**	CO_2_H (**1a**)	CH_3_CN	Et_3_N	10	49
12^b^^,^^d^	**4a**	CO_2_H (**1a**)	CH_2_Cl_2_	Et_3_N	10	27
13^b^^,^^d^	**4a**	CO_2_H (**1a**)	DMSO	Et_3_N	10	61
14^b^^,^^d^	**4a**	CO_2_H (**1a**)	DMF	Et_3_N	10	15
15^b^^,^^d^	**4a**	CO_2_H (**1a**)	CH_3_OH	Et_3_N	10	26
16^b^^,^^d^	**4a**	CO_2_H (**1a**)	DMSO	Et_3_N	5	67
17^b^^,^^d^	**4a**	CO_2_H (**1a**)	DMSO	Et_3_N	3	68
18^b^^,^^d^	**4a**	CO_2_H (**1a**)	DMSO	Et_3_N	1	47
19^b^^,^^d^	**4a**	CO_2_H (**1a**)	DMSO	DIPEA	3	42
20^b^^,^^d^	**4a**	CO_2_H (**1a**)	DMSO	DABCO	3	
21^b^^,^^d^	**4a**	CO_2_H (**1a**)	DMSO	NMM	3	49
22^b^^,^^d^^,^^e^	**4a**	CO_2_H (**1a**)	DMSO	Et_3_N	3	81
23^b^^,^^d^^,^^e^^,^^f^	**4a**	CO_2_H (**1a**)	DMSO	Et_3_N	3	93
24^b^^,^^d^^,^^e^^,^^f^^,^^g^	**4a**	CO_2_H (**1a**)	DMSO	Et_3_N	3	25
25^d^^,^^e^^,^^f^	**4a**	CO_2_H (**1a**)	DMSO	Et_3_N		
26^b^^,^^d^^,^^e^^,^^f^^,^^h^	**4a**	CO_2_H (**1a**)	DMSO	Et_3_N	3	
27^b^^,^^d^^,^^e^^,^^f^^,^^i^	**4a**	CO_2_H (**1a**)	DMSO	Et_3_N	3	74 (333 mg)
28^j^	**4a**	CO_2_H (**1a**)	DMSO	Et_3_N	3	

a All reactions were performed in a 0.1 mmol scale using **1a** or **5** (1.0 equiv) and **2a** (2.0 equiv) in the presence of the corresponding photoredox catalyst **4** (10 mol %) and the corresponding base (2.5 equiv) in the solvent (1 mL) for 24 h at room temperature.

b Reaction performed under irradiation with blue light.

c Reaction performed under irradiation with green light.

d Reaction performed using **2a** (3 equiv).

e Reaction performed for 48 h.

f Reaction performed in DMSO (3 mL).

g Reaction performed using Et_3_N (1 equiv).

h Reaction performed in the dark.

i Reaction performed at a 2 mmol scale.

j Reaction performed in the presence of TEMPO (1 equiv).

**Scheme 3:**
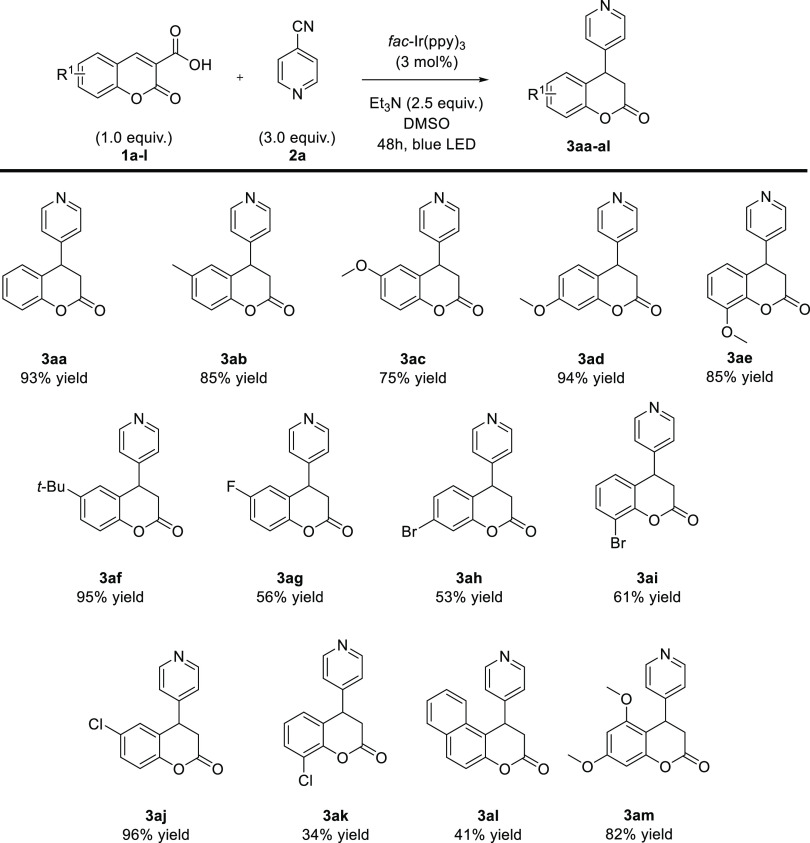
Visible Light-Driven Reductive Arylation of Coumarin-3-carboxylic Acids **1**: Scope of Coumarin-3-carboxylic Acids **1**

**Scheme 4:**
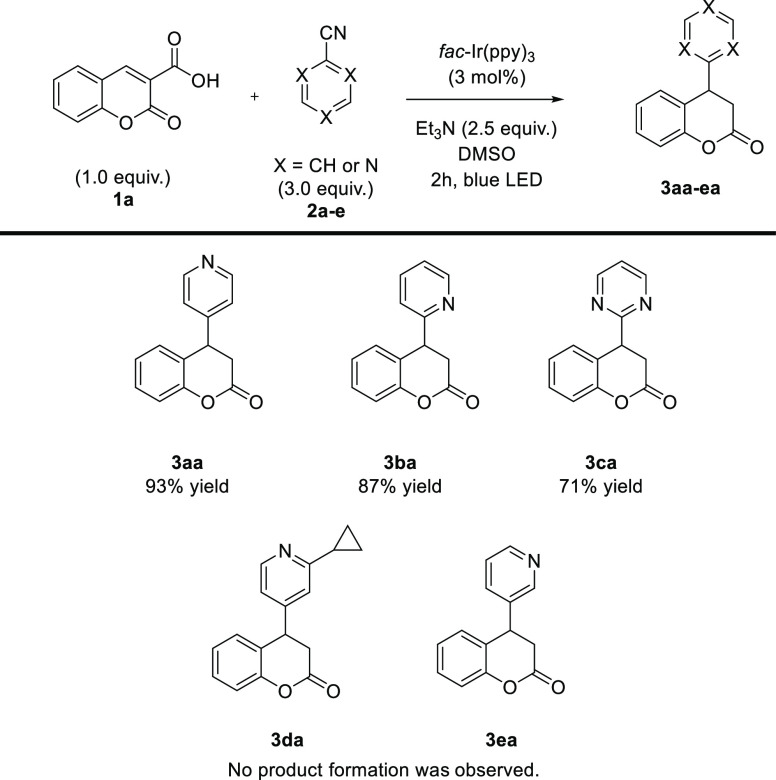
Visible Light-Driven Reductive Arylation of Coumarin-3-carboxylic Acids **1**: Reaction Involving Cyanoheteroaromatic Derivatives **2a**–**2c**

With the optimized reaction conditions in hand (Table 1, entry 23), the scope of the developed methodology was evaluated (Schemes 3 and 4). Initially, various coumarin-3-carboxylic acids **1a–m** were tested in the reaction (Scheme 3). Acids **1b–f** bearing electron-donating groups on the aromatic ring provided products **3ab**–**af** with very good yields. For the coumarin carboxylic acid **1a** with a *t*-butyl substituent at the 6-position of the aromatic ring, the yield was the highest despite the presence of a bulky *t*-butyl substituent. In the course of further studies, it was found that substrates **1** bearing electron-withdrawing groups delivered products **3** in diminished yields. Short reoptimization studies indicated that modification of a previously developed procedure (involving dropwise addition of coumarin carboxylic acids **1g–m** in dry DMSO (1 mL) over 2 h to the reaction mixture, see general procedure for details) enabled the improvement of the results. Dropwise addition of coumarin carboxylic acids **1g**–**m** suppressed its decomposition over reaction time. Under these conditions, the reaction using coumarins **1g–k** bearing fluorine, bromine, or chlorine atoms at various positions provided the corresponding products **3g–k** in moderate to high yields. It is only in the case of coumarin **1k** with a chlorine substituent in the 8-position of the aromatic ring that the yield of the reaction dropped to 34%. Similar results were observed for doubly substituted coumarin **1l**. In this context, it is worth noting that coumarin **1l** was not effective in the previous decarboxylative reactions performed by our group.^9d,10^ What is also worth emphasizing is that the reaction with doubly substituted coumarin **1m** with two methoxy substituents in the aromatic ring provided the desired product **3am** with very good yield.

Subsequently, the scope of the methodology with regard to different (cyano)azaarenes **2a**–**c** was evaluated (Scheme 4). It was demonstrated that the developed protocol worked well for 4- and 2-substituted pyridines **2a** and **2b** as well as pyrimidine-2-carbonitrile **3c** to give target products **3aa**–**3ca** with very good yields. Disappointingly, no product formation was observed when cyanopyridines **2d** and **2e** were employed under optimized reaction conditions.

**Scheme 5:**
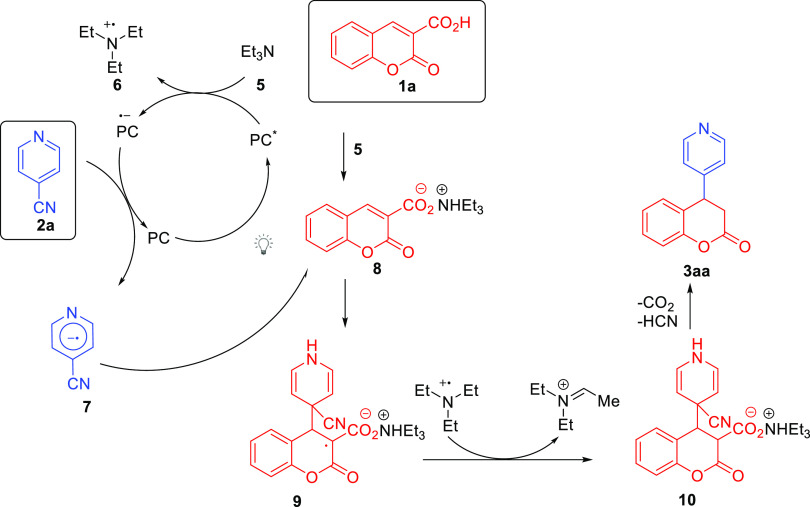
Visible Light-Driven Reductive Arylation of Coumarin-3-carboxylic Acids **1**: Reaction Mechanism

The postulated mechanism of the developed methodology begins with the blue light-driven excitation of the photocatalyst **4b** (Scheme 5). Then, the electron transfer from the triethylamine to the photocatalyst takes place. Fluorescence quenching and cyclic voltammetry experiments confirmed the lack of quenching in the case of acids **1a** as well as cyanopyridine **2a** (for details, see the Supporting Information). Subsequently, the reduced Ir-catalyst acts as a reductant of the (cyano)azaarene **2a** to give **7**. The newly formed radical **7** undergoes the decarboxylative Giese-type reaction with the acceptor **8** to give **9** that undergoes hydrogen atom transfer to give **10**. Two separate processes transform **10** into **3aa**: (1) rearomatization of the pyridine ring via dehydrocyanation and (2) decarboxylative protonation to afford **3aa** as the final product.

## Conclusions

In conclusion, we have developed a decarboxylative photocatalytic reductive arylation of coumarin-3-carboxylic acids **1** that represents a unique application of free-carboxylic-acid-activated olefins in radical transformations. The reactions between coumarin-3-carboxylic acids **1a**–**m** and (cyano)azaarenes **2a**–**c** were realized under photocatalytic activation in the presence of only 3 mol % of *fac*-Ir(ppy)_3_. The methodology proved versatile, leading to biologically relevant 4-substituted-chroman-2-ones **3aa**–**ca** in good to high yields under mild reaction conditions.

## Experimental Section

### General Information

NMR spectra were acquired on a Bruker Ultra Shield 700 instrument, running at 700 MHz for ^1^H and 176 MHz for ^13^C. Chemical shifts (δ) are reported in ppm relative to residual solvent signals (CDCl_3_: 7.26 ppm for ^1^H NMR, 77.16 ppm for ^13^C NMR). Mass spectra were recorded on a Bruker Maxis Impact spectrometer using electrospray (ES+) ionization (referenced to the mass of the charged species). Analytical thin layer chromatography (TLC) was performed using pre-coated aluminum-backed plates (Merck Kieselgel 60 F254) and visualized by ultraviolet irradiation. Unless otherwise noted, analytical-grade solvents and commercially available reagents were used without further purification. For flash chromatography (FC), silica gel (w/ Ca, ∼0.1%, 230–400 mesh), green LED (50 W, λ = 525 nm), and blue LED (50 W, λ = 456 nm) were purchased from commercial supplier Kessil LED Photoreactor Lightning. Fluorescence measurements were performed using a Varian Cary Eclipse spectrofluorometer equipped with a thermostatted cell holder. Coumarine-3-carboxylic acids **1b**–**k** were synthesized according to the literature procedure.^13^ Catalyst **4c** was synthesized according to the literature procedure.^14^

#### 6-Methyl-2-oxo-2*H*-chromene-3-carboxylic Acid (**1b**)

Compound **1b** was synthesized according to the literature procedure^13^ as a white solid in 75% yield (153.0 mg). Analytical data were in accordance with the literature.

#### 6-Methoxy-2-oxo-2*H*-chromene-3-carboxylic Acid (**1c**)

Compound **1c** was synthesized according to the literature procedure^13^ as a white solid in 82% yield (180.4 mg). Analytical data were in accordance with the literature.

#### 7-Methoxy-2-oxo-2*H*-chromene-3-carboxylic Acid (**1d**)

Compound **1d** was synthesized according to the literature procedure^13^ as a white solid in 64% yield (140.8 mg). Analytical data were in accordance with the literature.

#### 8-Methoxy-2-oxo-2*H*-chromene-3-carboxylic Acid (**1e**)

Compound **1e** was synthesized according to the literature procedure^13^ as a white solid in 72% yield (158.4 mg). Analytical data were in accordance with the literature.

#### 6-(*tert*-Butyl)-2-oxo-2*H*-chromene-3-carboxylic Acid (**1f**)

Compound **1f** was synthesized according to the literature procedure^13^ as a white solid in 89% yield (218.9 mg). Analytical data were in accordance with the literature.

#### 6-Fluoro-2-oxo-2*H*-chromene-3-carboxylic Acid (**1g**)

Compound **1g** was synthesized according to the literature procedure^13^ as a white solid in 84% yield (174.7 mg). Analytical data were in accordance with the literature.

#### 7-Bromo-2-oxo-2*H*-chromene-3-carboxylic Acid (**1h**)

Compound **1h** was synthesized according to the literature procedure^13^ as a yellow solid in 62% yield (166.8 mg). Analytical data were in accordance with the literature.

#### 8-Bromo-2-oxo-2*H*-chromene-3-carboxylic Acid (**1i**)

Compound **1i** was synthesized according to the literature procedure^13^ as a yellow solid in 54% yield (145.3 mg). Analytical data were in accordance with the literature.

#### 6-Chloro-2-oxo-2*H*-chromene-3-carboxylic Acid (**1j**)

Compound **1j** was synthesized according to the literature procedure^13^ as a yellow solid in 89% yield (199.8 mg). Analytical data were in accordance with the literature.

#### 8-Chloro-2-oxo-2*H*-chromene-3-carboxylic Acid (**1k**)

Compound **1k** was synthesized according to the literature procedure^13^ as a yellow solid in 72% yield (161.6 mg). Analytical data were in accordance with the literature.

#### 3-Oxo-3*H*-benzo[*f*]-chromene-2-carboxylic Acid (**1l**)

Compound **1l** was synthesized according to the literature procedure^13^ as a yellow solid in 67% yield (160.9 mg). Analytical data were in accordance with the literature.

#### 5,7-Dimethoxy-2-oxo-2*H*-chromene-3-carboxylic Acid (**1m**)

Compound **1m** was synthesized according to the literature procedure^13^ as a white solid in 56% yield (140.1 mg). Analytical data were in accordance with the literature.

#### General Procedure for the Synthesis of Substituted 4-(Pyridin-4-yl)chroman-2-ones (**3aa**–**3af**)

In a 10 mL Schlenk tube, coumarin-3-carboxylic acids **1a**–**f** (0.1 mmol, 1.0 equiv), 4-cyanopirydyne (0.3 mmol, 3.0 equiv), Et_3_N (0.25 mmol, 2.5 equiv), and catalyst *fac*-Ir(ppy)_3_ (3 mol %) were dissolved in dry DMSO (3 mL). The reaction mixture was degassed and filled three times with argon. Subsequently, the mixture was irradiated with blue LED for 48 h at room temperature. Next, the reaction was quenched with saturated solution of NaHCO_3_ (5 mL), extracted with CH_2_Cl_2_ (3 × 10 mL), and washed with brine (5 mL). The organic phase was dried over MgSO_4_ and concentrated under reduced pressure. The crude product was purified by silica gel chromatography (*n*-hexane:ethyl acetate, 2:1) to provide the desired products **3aa**–**af**.

#### 4-(Pyridin-4-yl)chroman-2-one (**3aa**)^15^

The pure product was isolated by flash chromatography on silica gel (*n*-hexane/ethyl acetate, 2:1) as a yellow oil in 93% yield (20.9 mg). ^1^H NMR (700 MHz, chloroform-*d*) δ 8.64–8.54 (m, 2H), 7.37–7.34 (m, 1H), 7.17 (dd, *J* = 8.2, 1.2 Hz, 1H), 7.14 (td, *J* = 7.5, 1.2 Hz, 1H), 7.10 (ddd, *J* = 4.4, 1.6, 0.6 Hz, 2H), 7.01 (dt, *J* = 7.5, 1.1 Hz, 1H), 4.34 (t, *J* = 6.5 Hz, 1H), 3.12 (dd, *J* = 16.0, 6.1 Hz, 1H), 3.04 (dd, *J* = 16.0, 6.8 Hz, 1H). ^13^C {^1^H} NMR (176 MHz, CDCl_3_) δ 166.7, 151.9, 150.7 (2C), 149.4, 129.6, 128.3, 125.1, 123.9, 122.7 (2C), 117.6, 40.2, 36.3. HRMS (ESI-TOF) *m/z* [M + H^+^] calculated for C_14_H_12_NO_2_^+^: 226.0863, found: 226.0864.

#### 6-Methyl-4-(pyridin-4-yl)chroman-2-one (**3ab**)

The pure product was isolated by flash chromatography on silica gel (*n*-hexane/ethyl acetate, 2:1) as a brown oil in 85% yield (20.3 mg). ^1^H NMR (700 MHz, chloroform-*d*) δ 8.59–8.56 (m, 2H), 7.13 (dd, *J* = 8.4, 2.1 Hz, 1H), 7.09–7.07 (m, 2H), 7.04 (d, *J* = 8.4 Hz, 1H), 6.79 (d, *J* = 2.1 Hz, 1H), 4.28 (t, *J* = 6.4 Hz, 1H), 3.08 (dd, *J* = 16.0, 6.2 Hz, 1H), 3.00 (dd, *J* = 16.0, 6.5 Hz, 1H), 2.61 (s, 1H), 2.28 (s, 3H). ^13^C {^1^H} NMR (176 MHz, CDCl_3_) δ 167.0, 150.7 (2C), 149.8, 149.5, 134.8, 130.1, 128.6, 123.5, 122.7 (2C), 117.3, 40.3, 36.4, 20.9. HRMS (ESI-TOF) *m/z* [M + H^+^] calculated for C_15_H_14_NO_2_^+^: 240.1019, found: 240.1016.

#### 6-Methoxy-4-(pyridin-4-yl)chroman-2-one (**3ac**)

The pure product was isolated by flash chromatography on silica gel (*n*-hexane/ethyl acetate, 2:1) as a yellow oil in 75% yield (19.1 mg). ^1^H NMR (700 MHz, chloroform-*d*) δ 8.60–8.55 (m, 2H), 7.11–7.08 (m, 3H), 6.87 (ddd, *J* = 8.9, 3.0, 0.5 Hz, 1H), 6.51 (dd, *J* = 3.0, 0.8 Hz, 1H), 4.28 (t, *J* = 6.4 Hz, 1H), 3.73 (s, 3H), 3.08 (dd, *J* = 16.0, 6.1 Hz, 1H), 3.00 (dd, *J* = 16.1, 6.7 Hz, 1H). ^13^C {^1^H} NMR (176 MHz, CDCl_3_) δ 166.9, 156.7, 150.6 (2C), 149.4, 145.8, 124.7, 122.8 (2C), 118.4, 114.5, 113.5, 55.8, 40.6, 36.3. HRMS (ESI-TOF) *m/z* [M + H^+^] calculated for C_15_H_14_NO_3_^+^: 256.0968, found: 256.0968.

#### 7-Methoxy-4-(pyridin-4-yl)chroman-2-one (**3ad**)

The pure product was isolated by flash chromatography on silica gel (*n*-hexane/ethyl acetate, 2:1) as a pale yellow oil in 94% yield (24.0 mg). ^1^H NMR (700 MHz, chloroform-*d*) δ 8.57 (d, *J* = 5.0 Hz, 2H), 7.09–7.07 (m, 2H), 6.89 (dd, *J* = 8.4, 0.8 Hz, 1H), 6.70 (d, *J* = 2.5 Hz, 1H), 6.67 (dd, *J* = 8.4, 2.6 Hz, 1H), 4.27 (t, *J* = 6.4 Hz, 1H), 3.81 (s, 3H), 3.09 (dd, *J* = 15.9, 6.2 Hz, 1H), 3.00 (dd, *J* = 15.9, 6.7 Hz, 1H). ^13^C {^1^H} NMR (176 MHz, CDCl_3_) δ 166.7, 160.6, 152.7, 150.5 (2C), 150.1, 128.9, 122.8 (2C), 115.6, 111.3, 103.0, 55.8, 39.7, 36.6. HRMS (ESI-TOF) *m/z* [M + H^+^] calculated for C_15_H_14_NO_3_^+^: 256.0968, found: 256.0964.

#### 8-Methoxy-4-(pyridin-4-yl)chroman-2-one (**3ae**)

The pure product was isolated by flash chromatography on silica gel (*n*-hexane/ethyl acetate, 2:1) as a pale yellow oil in 85% yield (21.7 mg). ^1^H NMR (700 MHz, chloroform-*d*) δ 8.57 (d, *J* = 5.0 Hz, 2H), 7.12–7.02 (m, 3H), 6.94 (dd, *J* = 8.2, 1.3 Hz, 1H), 6.59 (ddd, *J* = 7.7, 1.4, 0.8 Hz, 1H), 4.32 (t, *J* = 6.3 Hz, 1H), 3.92 (s, 3H), 3.14–3.07 (m, 1H), 3.03 (dd, *J* = 15.9, 6.3 Hz, 1H).^13^C {^1^H} NMR δ 166.1, 150.7 (2C), 149.3, 148.2, 141.2, 125.0, 124.9, 122.7 (2C), 119.6, 112.1, 56.3, 40.5, 36.1. HRMS (ESI-TOF) *m/z* [M + H^+^] calculated for C_15_H_14_NO_3_^+^: 256.0968, found: 256.0971.

#### 6-*tert*-Butyl-4-(pyridin-4-yl)chroman-2-one (**3af**)

The pure product was isolated by flash chromatography on silica gel (*n*-hexane/ethyl acetate, 2:1) as a yellow oil in 95% yield (26.7 mg). ^1^H NMR (700 MHz, chloroform-*d*) δ 8.58 (d, *J* = 5.1 Hz, 2H), 7.36 (dd, *J* = 8.6, 2.4 Hz, 1H), 7.10–7.08 (m, 3H), 7.02 (d, *J* = 2.4 Hz, 1H), 4.31 (t, *J* = 6.1 Hz, 1H), 3.10 (dd, *J* = 15.9, 6.2 Hz, 1H), 3.02 (dd, *J* = 15.9, 6.0 Hz, 1H), 1.25 (s, 9H). ^13^C {^1^H} NMR (176 MHz, CDCl_3_) δ 167.0, 150.7 (2C), 149.7, 149.7, 148.3, 126.6, 125.2, 122.9, 122.7 (2C), 117.1, 40.6, 36.6, 34.6, 31.5 (3C). HRMS (ESI-TOF) *m/z* [M + H^+^] calculated for C_18_H_20_NO_2_^+^: 282.1489, found: 282.1491.

#### General Procedure for the Synthesis of Substituted 4-(Pyridin-4-yl)chroman-2-one **3ag–am**

In a 10 mL Schlenk tube, 4-cyanopirydyne **2a** (0.3 mmol, 3.0 equiv), Et_3_N (0.25 mmol, 2.5 equiv), and catalyst *fac*-Ir(ppy)_3_ (3 mol %) were dissolved in dry DMSO (2 mL). The reaction mixture was degassed and filled three times with argon. The mixture was irradiated with blue LED for 2 h at room temperature. Subsequently, coumarin-3-carboxylic acids **1g**–**m** (0.1 mmol, 1.0 equiv) in dry DMSO (1 mL) was added dropwise over 2 h and stirred for additional 48 h. Next, the reaction was quenched with saturated solution of NaHCO_3_ (5 mL), extracted with CH_2_Cl_2_ (3 × 10 mL), and washed with brine (5 mL). The organic phase was dried over MgSO_4_ and concentrated under reduced pressure. The crude product was purified by silica gel chromatography (*n*-hexane:ethyl acetate, 2:1) to provide the desired products **3ag**–**am**.

#### 6-Fluoro-4-(pyridin-4-yl)chroman-2-one (**3ag**)

The pure product was isolated by flash chromatography on silica gel (*n*-hexane/ethyl acetate, 2:1) as a yellow oil in 56% yield (13.6 mg). ^1^H NMR (700 MHz, chloroform-*d*) δ 8.62–8.60 (m, 2H), 7.13 (dd, *J* = 9.0, 4.6 Hz, 1H), 7.10–7.08 (m, 2H), 7.04 (ddd, *J* = 9.0, 7.7, 2.9 Hz, 1H), 6.70 (ddd, *J* = 8.2, 2.9, 0.8 Hz, 1H), 4.31 (t, *J* = 6.7 Hz, 1H), 3.09 (dd, *J* = 16.0, 6.1 Hz, 1H), 3.02 (dd, *J* = 16.0, 7.3 Hz, 1H). ^13^C {^1^H} NMR (176 MHz, chloroform-*d*) δ 166.3, 159.3 (d, *J* = 245.2 Hz), 150.9 (2C), 148.5, 147.9 (d, *J* = 2.8 Hz), 125.6 (d, *J* = 7.6 Hz), 122.7 (2C), 119.0 (d, *J* = 8.3 Hz), 116.4 (d, *J* = 23.5 Hz), 114.9 (d, *J* = 24.4 Hz), 40.3, 35.9. HRMS (ESI-TOF) *m/z* [M + H^+^] calculated for C_14_H_11_NO_2_F ^+^: 244.0768, found: 244.0769.

#### 7-Bromo-4-(pyridin-4-yl)chroman-2-one (**3ah**)

The pure product was isolated by flash chromatography on silica gel (*n*-hexane/ethyl acetate, 2:1) as a pale yellow oil in 53% yield (16.1 mg). ^1^H NMR (700 MHz, chloroform-*d*) δ 8.61–8.60 (m, 2H), 7.34 (d, *J* = 2.0 Hz, 1H), 7.28–7.25 (m, 1H), 7.09–7.07 (m, 2H), 6.88 (d, *J* = 8.1 Hz, 1H), 4.29 (t, *J* = 6.6 Hz, 1H), 3.10 (dd, *J* = 16.0, 6.1 Hz, 1H), 3.02 (dd, *J* = 16.0, 7.0 Hz, 1H). ^13^C {^1^H} NMR (176 MHz, CDCl_3_) δ 165.8, 152.4, 150.9 (2C), 148.7, 129.5, 128.2, 123.0, 122.6 (2C), 122.6, 126.0, 77.2, 40.0, 36.1. HRMS (ESI-TOF) *m/z* [M + H^+^]calculated for C_14_H_11_BrNO_2_^+^: 303.9968, found: 303.9971.

#### 8-Bromo-4-(pyridin-4-yl)chroman-2-one (**3ai**)

The pure product was isolated by flash chromatography on silica gel (*n*-hexane/ethyl acetate, 2:1) as a pale yellow oil in 61% yield (18.5 mg). ^1^H NMR (700 MHz, chloroform-*d*) δ 8.62–8.58 (m, 2H), 7.59 (ddd, *J* = 8.0, 1.6, 0.5 Hz, 1H), 7.08 (ddd, *J* = 4.4, 1.7, 0.6 Hz, 2H), 7.01 (t, *J* = 7.6 Hz, 1H), 6.95 (ddd, *J* = 7.6, 1.6, 0.8 Hz, 1H), 4.35 (t, *J* = 6.5 Hz, 1H), 3.12 (dd, *J* = 15.9, 6.1 Hz, 1H), 3.07 (dd, *J* = 15.9, 6.9 Hz, 1H). ^13^C {^1^H} NMR (176 MHz, CDCl_3_) δ 165.4, 150.9 (2C), 148.9, 148.6, 133.5, 127.4, 125.8, 125.7, 122.6 (2C), 111.5, 40.6, 36.0. HRMS (ESI-TOF) *m/z* [M + H^+^] calculated for C_14_H_11_NO_2_Br ^+^: 303.9968, found: 303.9973.

#### 6-Chloro-4-(pyridin-4-yl)chroman-2-one (**3aj**)

The pure product was isolated by flash chromatography on silica gel (*n*-hexane/ethyl acetate, 2:1) as a pale yellow oil in 96% yield (24.9 mg). ^1^H NMR (700 MHz, chloroform-*d*) δ 8.63–8.61 (m, 2H), 7.32 (ddd, *J* = 8.7, 2.5, 0.6 Hz, 1H), 7.11 (d, *J* = 8.7 Hz, 1H), 7.09 (ddd, *J* = 4.4, 1.6, 0.6 Hz, 2H), 6.98 (dd, *J* = 2.5, 0.8 Hz, 1H), 4.31 (t, *J* = 6.6 Hz, 1H), 3.10 (dd, *J* = 16.0, 6.1 Hz, 1H), 3.03 (dd, *J* = 16.0, 7.1 Hz, 1H). ^13^C {^1^H} NMR (176 MHz, CDCl_3_) δ 166.0, 150.9 (2C), 150.4, 148.4, 130.3, 129.7, 128.2, 125.6, 122.6 (2C), 119.0, 40.2, 35.9. HRMS (ESI-TOF) *m/z* [M + H^+^] calculated for C_14_H_11_ClNO_2_^+^: 260.0473, found: 260.0471.

#### 8-Chloro-4-(pyridin-4-yl)chroman-2-one (**3ak**)

The pure product was isolated by flash chromatography on silica gel (*n*-hexane/ethyl acetate, 2:1) as a pale yellow oil in 34% yield (8.8 mg). ^1^H NMR (700 MHz, chloroform-*d*) δ 8.60 (d, *J* = 5.0 Hz, 2H), 7.42 (d, *J* = 8.0 Hz, 1H), 7.07 (dd, *J* = 21.2, 6.4 Hz, 3H), 6.91 (d, *J* = 7.7 Hz, 1H), 4.36 (t, *J* = 6.6 Hz, 1H), 3.12 (dd, *J* = 15.9, 6.1 Hz, 1H), 3.07 (dd, *J* = 15.9, 6.9 Hz, 1H). ^13^C {^1^H} NMR (176 MHz, CDCl_3_) δ 165.4, 150.8 (2C), 148.7, 147.8, 130.5, 126.6, 125.8, 125.2, 122.8, 122.7 (2C), 40.6, 36.0. HRMS (ESI-TOF) *m/z* [M + H^+^] calculated for C_14_H_11_ClNO_2_^+^: 260.0473, found: 260.0475.

#### 1,2-Dihydro-3*H*-benzo[*f*]-1-(pyridin-4-yl)chromen-3-one (**3al**)^16^

The pure product was isolated by flash chromatography on silica gel (*n*-hexane/ethyl acetate, 2:1) as a pale yellow oil in 41% yield (11.3 mg). ^1^H NMR (700 MHz, chloroform-*d*) δ 8.53–8.51 (m, 2H), 7.92 (d, *J* = 9.1 Hz, 1H), 7.90–7.89 (m, 1H), 7.71 (dd, *J* = 8.5, 0.9 Hz, 1H), 7.51 (ddd, *J* = 8.5, 6.8, 1.4 Hz, 1H), 7.48 (ddd, *J* = 8.0, 6.8, 1.6 Hz, 1H), 7.37 (d, *J* = 9.0 Hz, 1H), 7.06 (ddd, *J* = 4.4, 1.6, 0.6 Hz, 2H), 4.94 (d, *J* = 6.7 Hz, 1H), 3.27 (dd, *J* = 16.0, 7.3 Hz, 1H), 3.18 (dd, *J* = 16.0, 1.8 Hz, 1H). ^13^C {^1^H} NMR (176 MHz, CDCl_3_) δ 166.3, 150.8 (2C), 150.1, 149.4, 131.3, 130.9, 130.7, 129.1, 128.0, 125.7, 122.7, 122.3 (2C), 117.7, 116.0, 37.1, 36.6. HRMS (ESI-TOF) *m/z* [M + H^+^] calculated for C_18_H_14_NO_2_^+^: 276.1019, found: 276.1023.

#### 5,7-Dimethoxy-4-(pyridin-4-yl)chroman-2-one (**3am**)

The pure product was isolated by flash chromatography on silica gel (*n*-hexane/ethyl acetate, 2:1) as a pale yellow oil in 82% yield (23.4 mg). ^1^H NMR (700 MHz, chloroform-*d*) δ 8.63–8.29 (m, 2H), 7.17–6.90 (m, 2H), 6.31 (d, *J* = 2.3 Hz, 1H), 6.28 (d, *J* = 2.3 Hz, 1H), 4.52 (dd, *J* = 6.8, 2.4 Hz, 1H), 3.80 (s, 3H), 3.75 (s, 3H), 3.08–2.96 (m, 2H). ^13^C {^1^H} NMR (176 MHz, CDCl_3_) δ 166.9, 161.3, 157.5, 153.2, 150.6, 150.3 (3C), 122.2, 104.4, 95.3, 94.3, 56.0, 55.7, 36.0, 34.1. HRMS (ESI-TOF) *m/z* [M + H^+^] calculated for C_16_H_16_NO_4_^+^: 286.1074, found: 286.1068.

#### General Procedure for the Synthesis of 4-Substituted-chroman-2-one **3ba** and **3ca**

In a 10 mL Schlenk tube, Et_3_N (0.25 mmol, 2.5 equiv) and catalyst *fac*-Ir(ppy)_3_ (3 mol %) were dissolved in dry DMSO (1 mL). The reaction mixture was degassed and filled three times with argon. The mixture was irradiated with blue LED at room temperature. Coumarin-3-carboxylic acid **1a** (0.1 mmol, 1.0 equiv) in dry DMSO (1 mL) and cyanoarenes **2a**–**c** (0.3 mmol, 3.0 equiv) in dry DMSO (1 mL) were added dropwise over 2 h and stirred for additional 48 h. Next, the reaction was quenched with saturated solution of NaHCO_3_ (5 mL), extracted with CH_2_Cl_2_ (3 × 10 mL), and washed with brine (5 mL). The organic phase was dried over MgSO_4_ and concentrated under reduced pressure. The crude product was purified by silica gel chromatography (*n*-hexane:ethyl acetate, 5:1) to provide the desired products **3ba** and **3ca**.

#### 4-(Pyridin-2-yl)chroman-2-one (**3ba**)

The pure product was isolated by flash chromatography on silica gel (*n*-hexane/ethyl acetate, 10:1) as a pale yellow oil in 87% yield (19.8 mg). ^1^H NMR (700 MHz, chloroform-*d*) δ 8.57 (ddd, *J* = 4.8, 1.9, 1.0 Hz, 1H), 7.62 (td, *J* = 7.7, 1.9 Hz, 1H), 7.29–7.27 (m, 1H), 7.17 (ddd, *J* = 7.6, 4.8, 1.1 Hz, 1H), 7.14–7.10 (m, 2H), 7.10–7.06 (m, 2H), 4.43 (dd, *J* = 6.3, 5.1 Hz, 1H), 3.31 (dd, *J* = 16.0, 5.1 Hz, 1H), 3.04 (dd, *J* = 16.0, 6.3 Hz, 1H). ^13^C {^1^H} NMR (176 MHz, CDCl_3_) δ 167.9, 160.1, 151.8, 150.2, 137.1, 129.1, 128.3, 124.6 (2C), 122.6, 122.0, 117.6, 43.0, 34.8. HRMS (ESI-TOF) *m/z* [M + H^+^] calculated for C_14_H_12_NO_2_^+^: 226.0863, found: 226.0864.

#### 4-(Pyrimidin-2-yl)chroman-2-one (**3ca**)

The pure product was isolated by flash chromatography on silica gel (*n*-hexane/ethyl acetate, 10:1) as a yellow oil in 71% yield (16.0 mg). ^1^H NMR (700 MHz, chloroform-*d*) δ 8.67 (d, *J* = 4.9 Hz, 2H), 7.34 (ddd, *J* = 7.9, 1.6, 0.7 Hz, 1H), 7.28–7.25 (m, 1H), 7.16 (t, *J* = 4.9 Hz, 1H), 7.10–7.07 (m, 2H), 4.59 (dd, *J* = 6.5, 3.2 Hz, 1H), 3.29 (dd, *J* = 16.1, 3.2 Hz, 1H), 3.07 (dd, *J* = 16.1, 6.5 Hz, 1H). ^13^C {^1^H} NMR (176 MHz, CDCl_3_) δ 169.8, 167.7, 157.7 (2C), 151.6, 129.3, 128.7, 124.6, 123.3, 119.7, 117.6, 44.5, 33.6. HRMS (ESI-TOF) *m/z* [M + H^+^] calculated for C_13_H_11_N_2_O_2_^+^: 227.0815, found: 227.0818.

#### General Procedure for the Synthesis of 4-(Pyridin-4-yl)chroman-2-one (**3aa**) in a 2 mmol Scale^15^

In a 50 mL Schlenk tube, coumarin-3-carboxylic acid **1a** (380.3 mg, 2.0 mmol, 1.0 equiv), 4-cyanopirydyne **2a** (624.7 mg, 6.0 mmol, 3.0 equiv), Et_3_N (506.0 mg, 5.0 mmol, 2.5 equiv), and catalyst *fac*-Ir(ppy)_3_ (39.3 mg, 3 mol %) were dissolved in dry DMSO (20 mL). The reaction mixture was degassed and filled three times with argon. Subsequently, the mixture was irradiated with blue LED for 48 h at room temperature. Next, the reaction was quenched with saturated solution of NaHCO_3_ (50 mL), extracted with CH_2_Cl_2_ (3 × 75 mL), and washed with brine (50 mL). The organic phase was dried over MgSO_4_ and concentrated under reduced pressure. The crude product **3aa** was purified by silica gel chromatography (*n*-hexane:ethyl acetate, 2:1) to provide the desired product **3aa** as a yellow oil in 74% yield (333 mg).

## Supporting Information Available

Cyclic voltammetry, fluorescence quenching, photochemical reaction setup, and copies of ^1^H and ^13^C NMR spectra (PDF)
