# Supplementary material for: Visible Light-Driven Reductive Azaarylation of Coumarin-3-carboxylic Acids
Source: J Org Chem. 2022 Jul 12;87(15):9645–53. doi: 10.1021/acs.joc.2c00683 (PMC9361294; doi:10.1021/acs.joc.2c00683)

Visible-light driven reductive azaarylation of coumarin-3-carboxylic acids

Ewelina Kowalska,[a] Angelika Artelska[b] and Anna Albrecht[c*]

[a] Institute of Organic Chemistry, Lodz University of Technology, Żeromskiego 116, 90-924 Łódź, Poland

[b] Institute of Applied Radiation Chemistry, Lodz University of Technology, Żeromskiego 116, 90-924 Łódź, Poland

[c] Institute of General and Ecological Chemistry, Faculty of Chemistry, Lodz University of Technology, Żeromskiego 116, 90-924 Łódź, Poland

anna.albrecht@p.lodz.pl

Contents

1. General methods S2

2. Cyclic voltammetry S4

3. Fluorescence Quenching S6

4. NMR data S8

1. General methods

NMR spectra were acquired on a Bruker Ultra Shield 700 instrument, running at 700 MHz for ^{1}H and 176 MHz for ^{13}C, respectively. Chemical shifts (δ) are reported in ppm relative to residual solvent signals ($CDCl_3$: 7.26 ppm for ^{1}H NMR, 77.16 ppm for ^{13}C {^{1}H} NMR. Mass spectra were recorded on a Bruker Maxis Impact spectrometer using electrospray (ES+) ionization (referenced to the mass of the charged species). Analytical thin layer chromatography (TLC) was performed using pre-coated aluminum-backed plates (Merck Kieselgel 60 F254) and visualized by ultraviolet irradiation. Unless otherwise noted, analytical grade solvents and commercially available reagents were used without further purification. For flash chromatography (FC) silica gel (Silica gel, w/Ca, ~0.1%), 230-400 mesh). Green LED (50 W, λ = 525 nm), blue LED (50 W, λ = 456 nm), were purchased from commercial supplier Kessil LED photoreactor lightning. Fluorescence measurements were performed using Varian Cary Eclipse spectrofluorometer equipped with thermos stated cell holder. Coumarine-3-carboxylic acids[1] **1b-m** were synthetized according to the literature procedure. Catalyst **4c** was synthesized according to the literature procedure.[2]

Figure S1 shows the 50W 456 nm photochemical reaction setup. The reaction vials in front of the 50W 456 nm bulb at approximatively 4.5 cm distance. To maintain a stable reaction temperature two fans were placed in close proximity to the reaction vials (23±2 °C).

[1] A. Song, X. Wang, K. S. Lam, *Tetrahedron Lett*. **2003**, *44*, 1755.
[2] E. Speckmeier, T. G. Fischer, K. Zeitler *J. Am. Chem. Soc.* **2018**, *140*, 45, 15353.

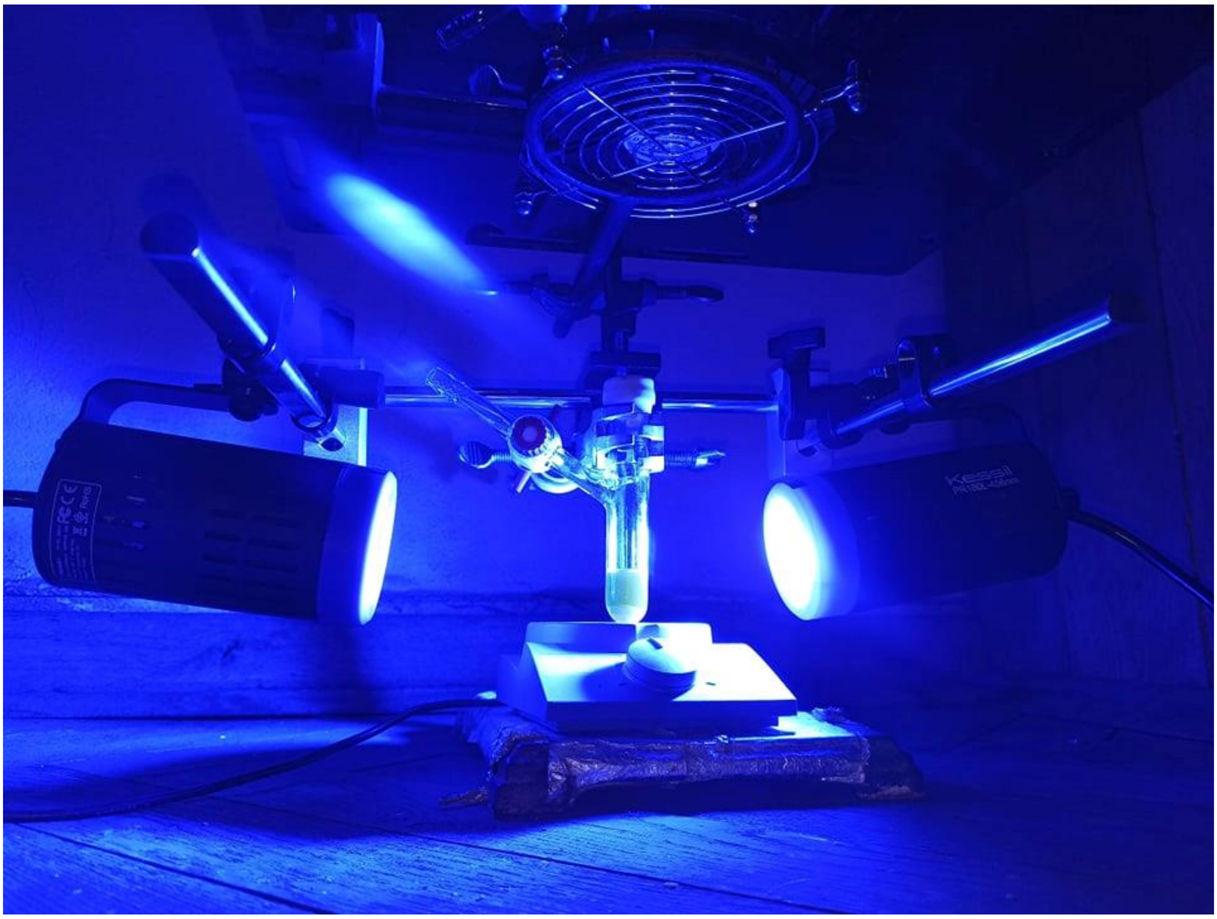

Fig. S1 Photochemical reaction setup using 50W 456nm Kessil LED.

2. Cyclic voltammetry

The electrochemical measurements were carried out in a three-electrode electrochemical cell using Autolab PGSTAT302N potentiostat/galvanostat (Metrohm). Platinum plate was applied as a working electrode. Its potential was measured vs. saturated calomel electrode (SCE) as a reference one. Platinum wire was an auxiliary electrode.

All measurements were carried out in CH_3CN with 0.1 mol/L tetrabutylammonium perchlorate (TBAP) as the supporting electrolyte under room temperature. The solutions were degassed with argon prior to measurements. The concentration of substrates was 1 mmol/L. Cyclic voltammograms were recorded with the scan rate of 0.1 V/s.

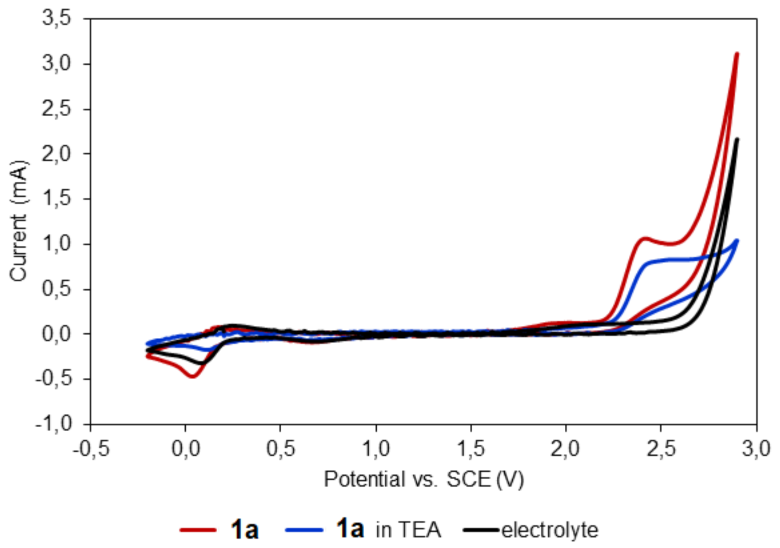

Fig. S2 Cyclic voltammograms recorded in coumarin-3-carboxylic acid **1a** solution compound in the presence and without triethylamine, and in supporting electrolyte tetrabutylammonium perchlorate (TBAP) in CH_3CN; v = 0.1 V/s.

$E_{1/2}$ values determined for coumarin-3-carboxylic acid **1a** vs. SCE (saturated calomel electrode)

coumarin-3-carboxylic acid **1a** – $E_{1/2}$ = 2.357 V,

coumarin-3-carboxylic acid **1a** in triethylamine – $E_{1/2}$ = 2.392 V.

Coumarin-3-carboxylic acid **1a** is oxidized at the Pt electrode in at least one electrode step in the potential range from 2.2 to 2.9 V. In the presence of triethylamine, the oxidation of coumarin-3-carboxylic acid **1a** proceeds in at least two electrode steps. The half-wave potential determined for the first step of coumarin-3-carboxylic acid **1a** oxidation is by 35 mV higher in

the presence of triethylamine. This implies a hindrance of coumarin-3-carboxylic acid **1a** oxidation caused by triethylamine presence. Furthermore, a clear decrease in the peak current related to the first step of coumarin-3-carboxylic acid **1a** oxidation, is observed in the presence of triethylamine. This suggests that the oxidation of coumarin-3-carboxylic acid **1a** proceeds with higher rate in the solution without triethylamine.

3. Fluorescence Quenching

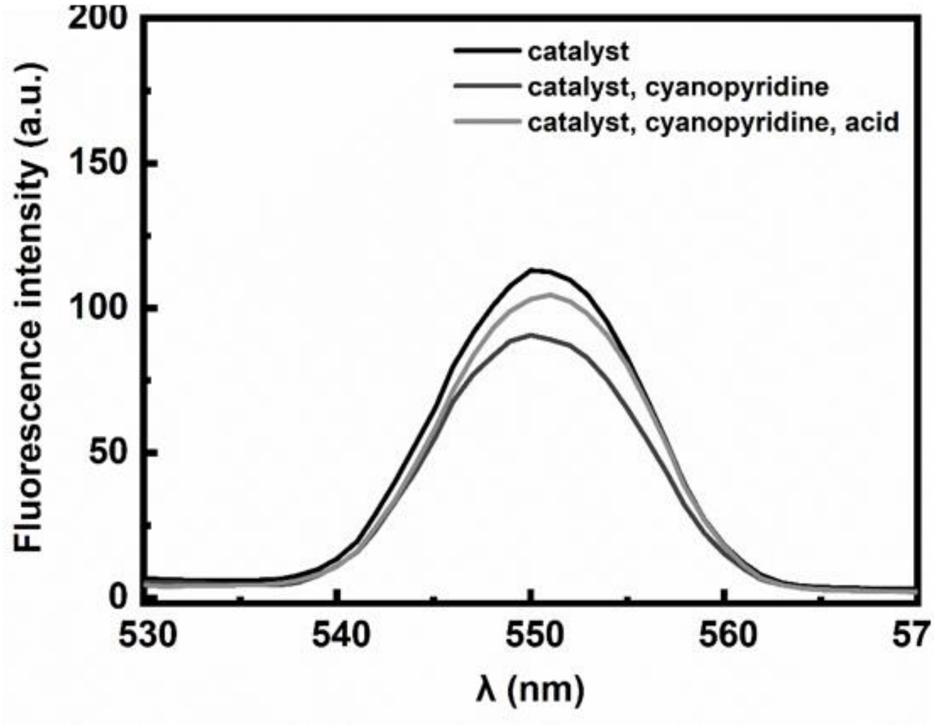

Fig. S3 Fluoresence spectra of *fac*Ir(ppy)$_3$ (25 μM) in CH_2Cl_2, *fac*Ir(ppy)$_3$ (25 μM) and 4-cyanopyridine (200 μM) in CH_2Cl_2, *fac*Ir(ppy)$_3$ (25 μM), 4-cyanopyridine **2a** (200 μM) and coumarin-3- carboxylic acid **1a** (66 μM) in CH_2Cl_2. Excitation and emission wavelength were 275 nm and 551 nm.

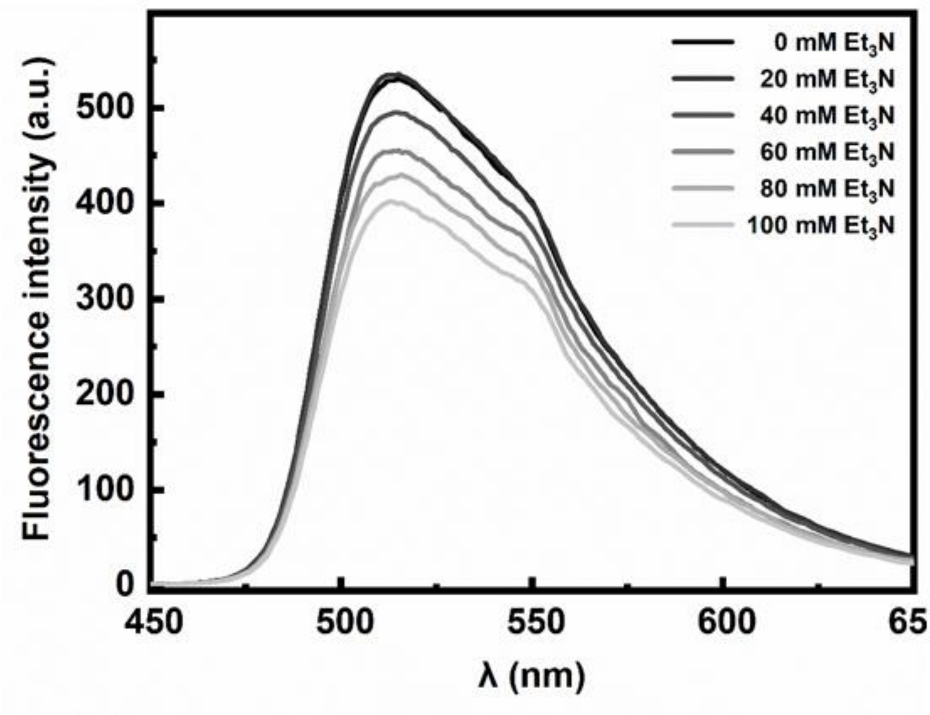

Fig. S4 Fluorescence quenching of *fac*Ir(ppy)$_3$ (25 μM) by Et_3N in CH_2Cl_2. Excitation and emission wavelength were 275 nm and 551 nm.

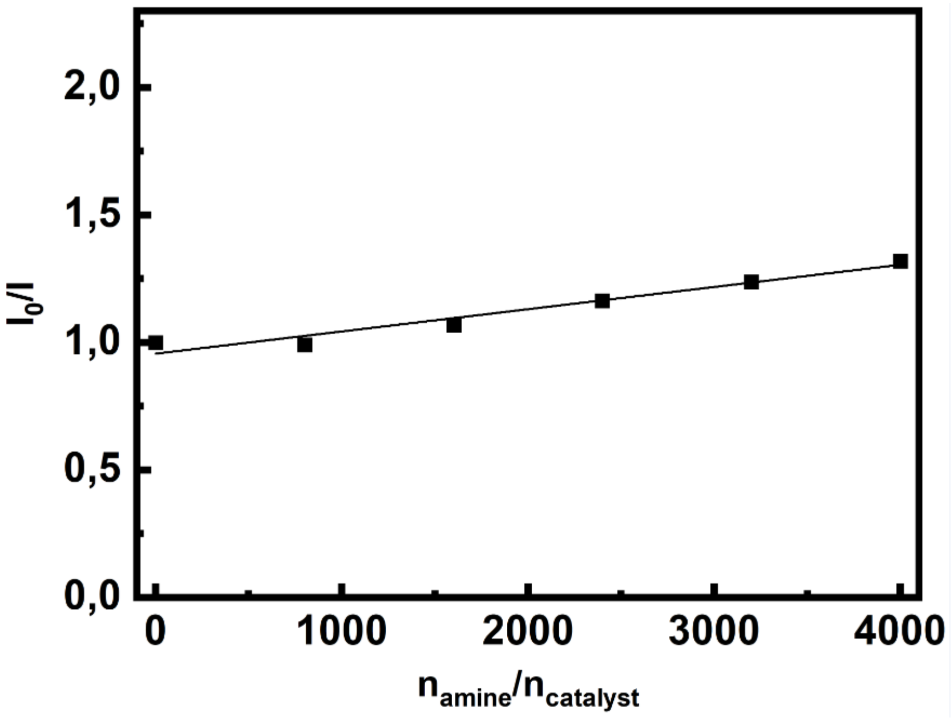

Fig. S5 Stern-Volmer plot of Fluorensence quenching of *fac*Ir(ppy)$_3$ by Et_3N.

4. NMR Data

4-(Pyridin-4-yl)chroman-2-one 3aa

^{1}H NMR ($CDCl_3$, 700 MHz)

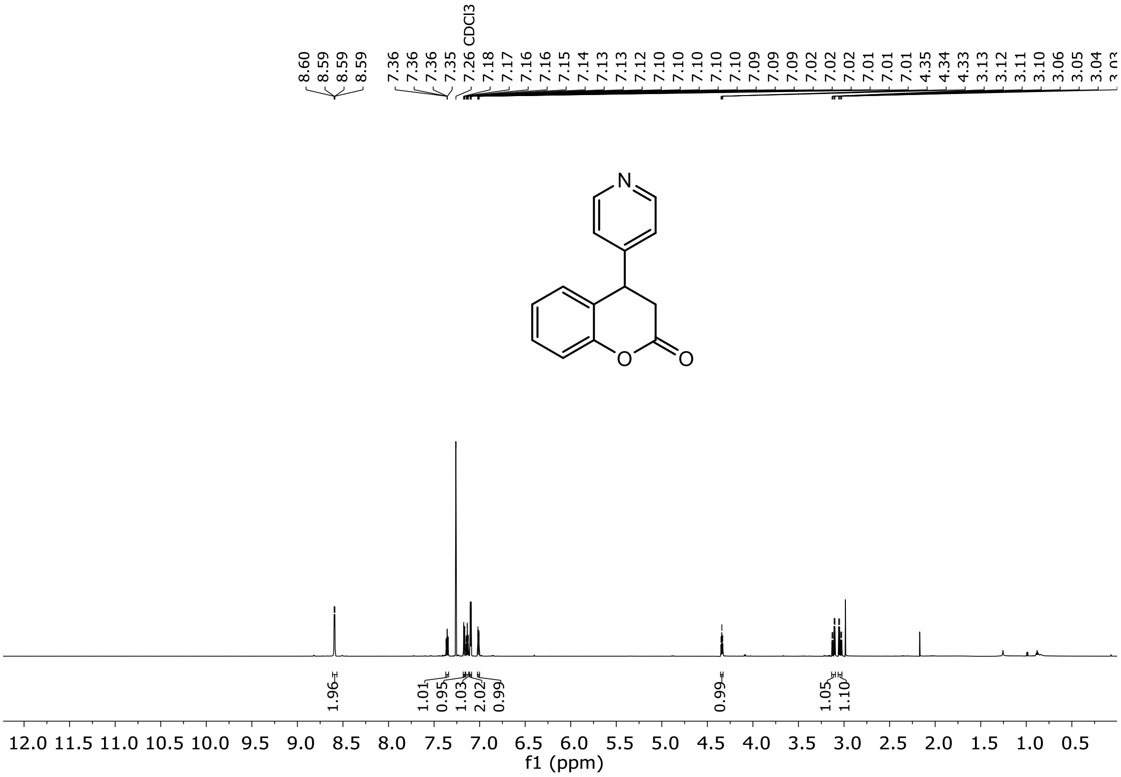

^{13}C {^{1}H} NMR ($CDCl_3$, 176 MHz)

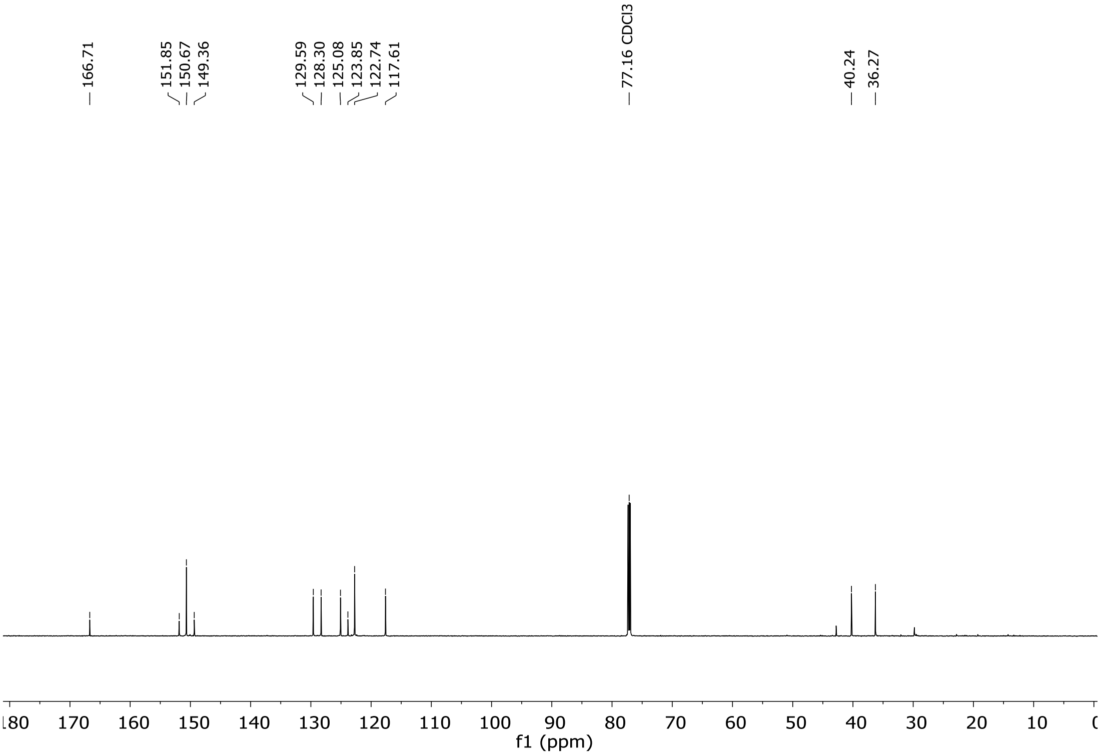

6-Methyl-4-(pyridin-4-yl)chroman-2-one 3ab

^{1}H NMR ($CDCl_3$, 700 MHz)

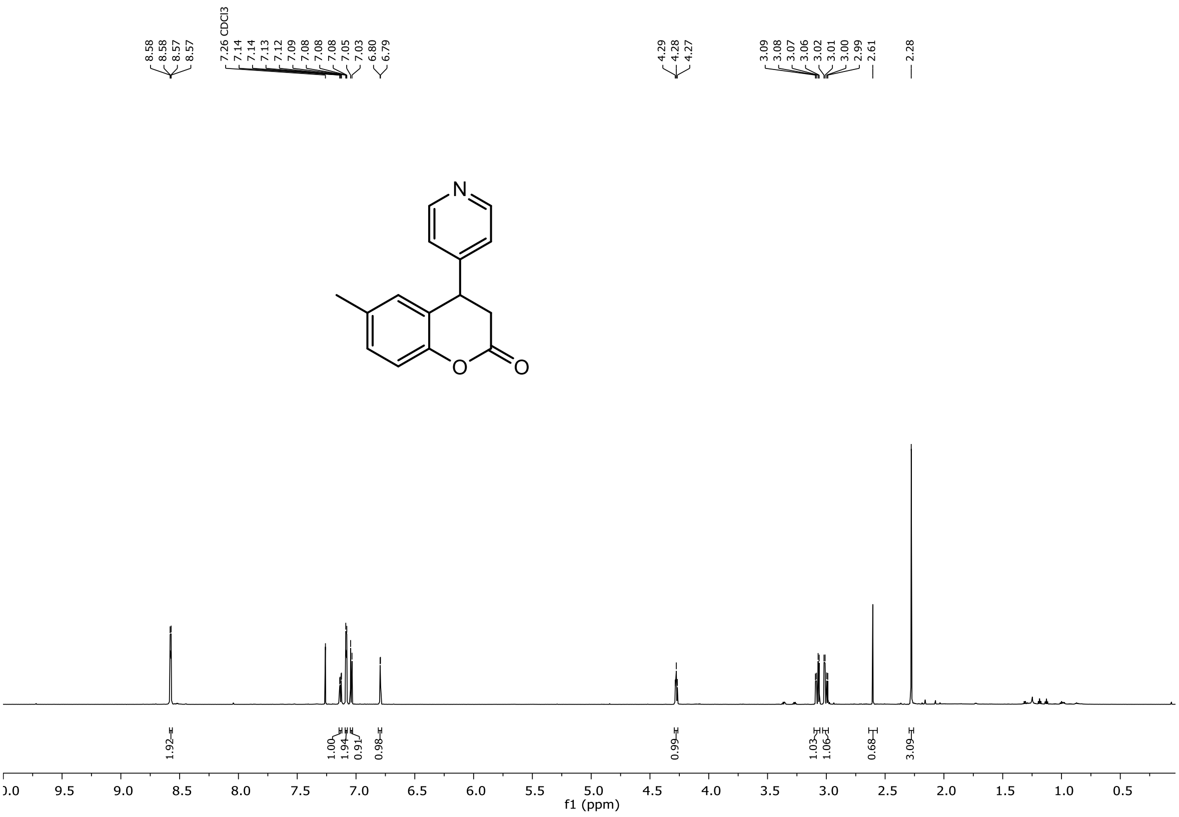

^{13}C {^{1}H} NMR ($CDCl_3$, 176 MHz)

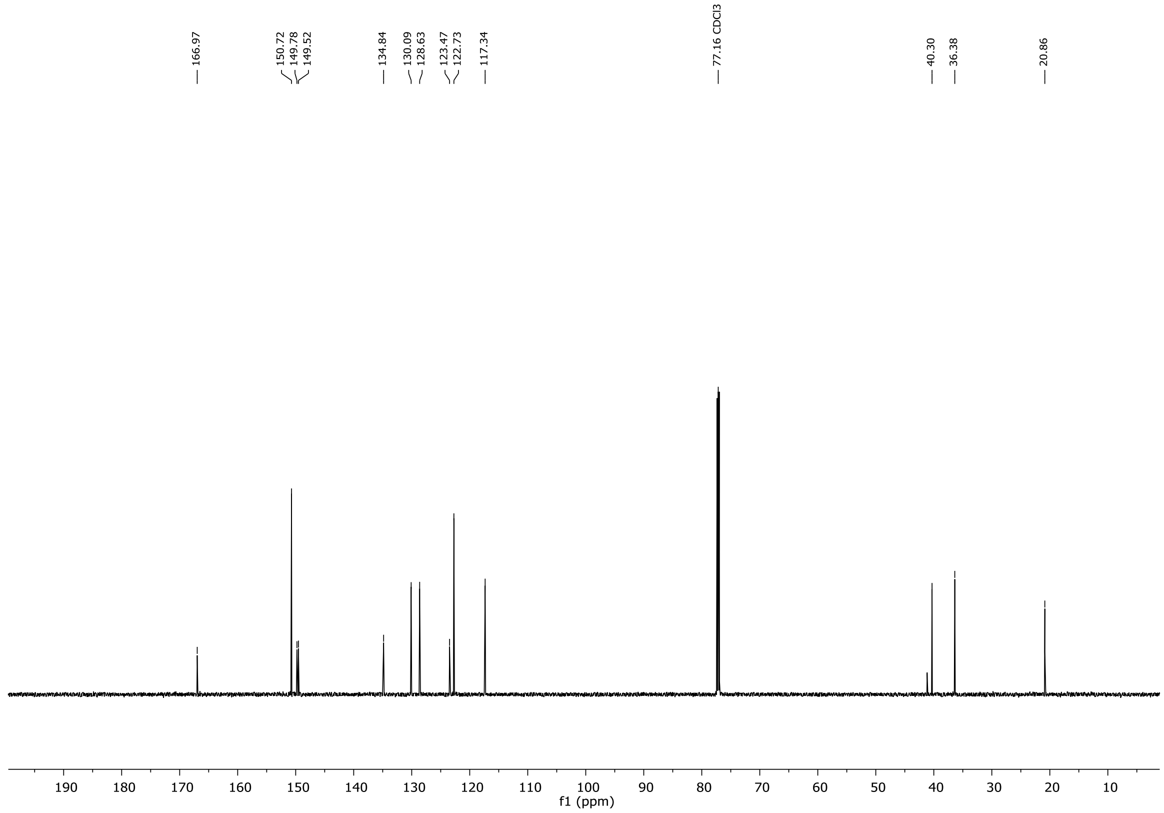

6-Methoxy-4-(pyridin-4-yl)chroman-2-one 3ac

^{1}H NMR ($CDCl_3$, 700 MHz)

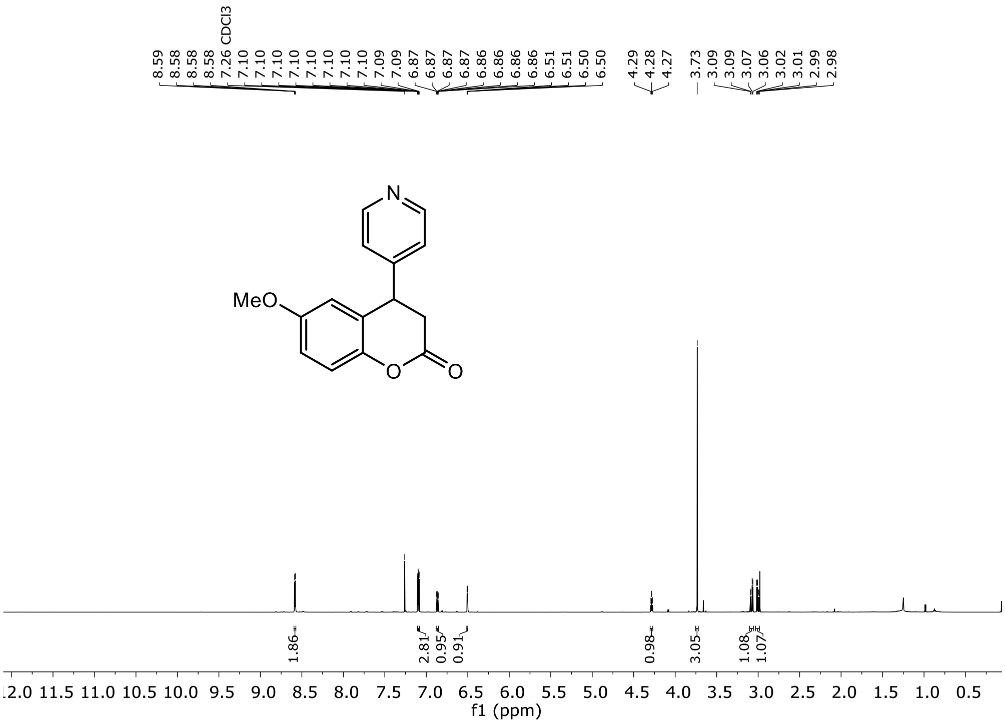

^{13}C {^{1}H} NMR ($CDCl_3$, 176 MHz)

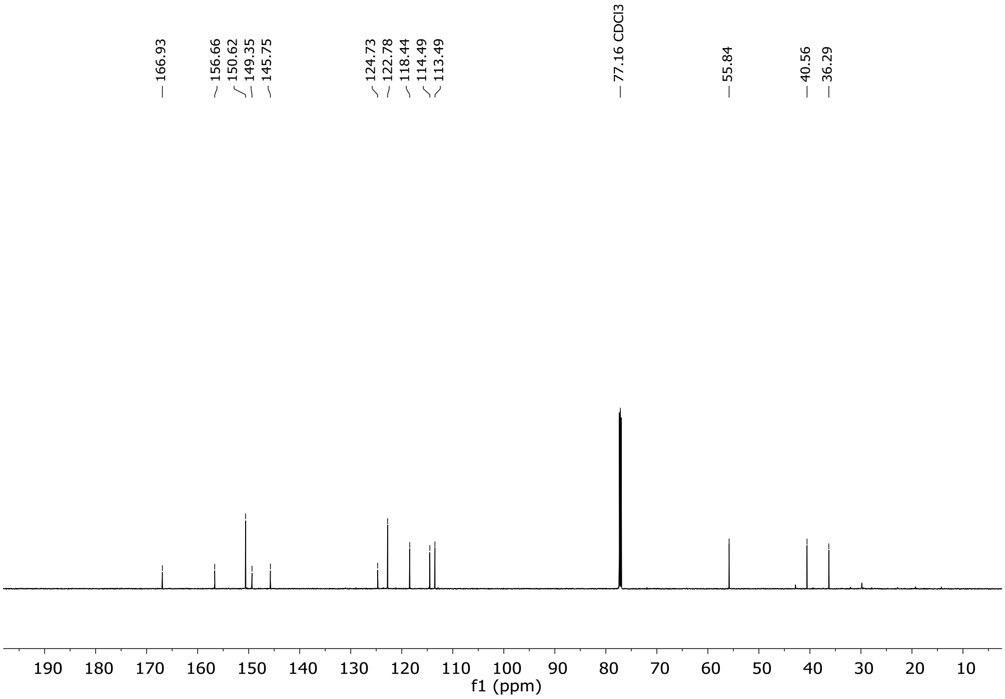

7-Methoxy-4-(pyridin-4-yl)chroman-2-one 3ad

^{1}H NMR ($CDCl_3$, 700 MHz)

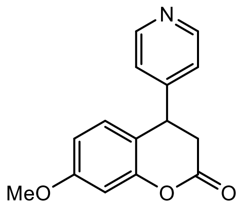

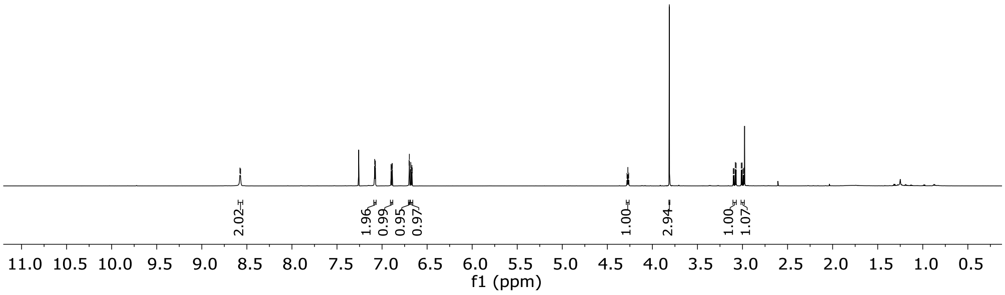

^{13}C {^{1}H} NMR ($CDCl_3$, 176 MHz)

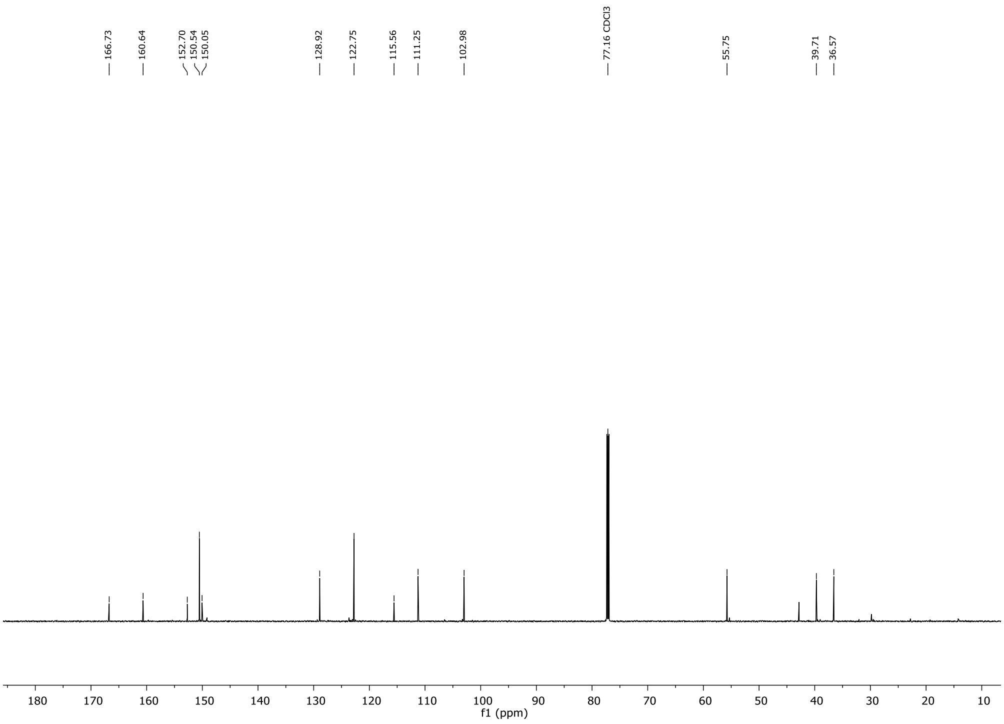

8-Methoxy-4-(pyridin-4-yl)chroman-2-one 3ae

^{1}H NMR ($CDCl_3$, 700 MHz)

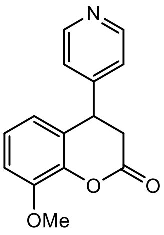

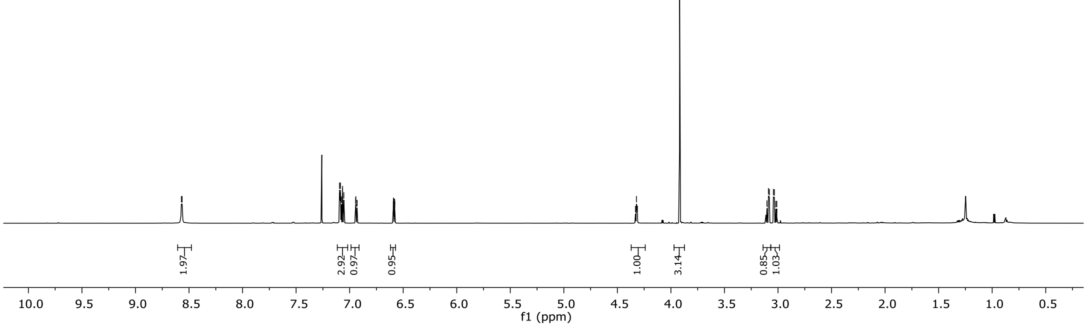

^{13}C {^{1}H} NMR ($CDCl_3$, 176 MHz)

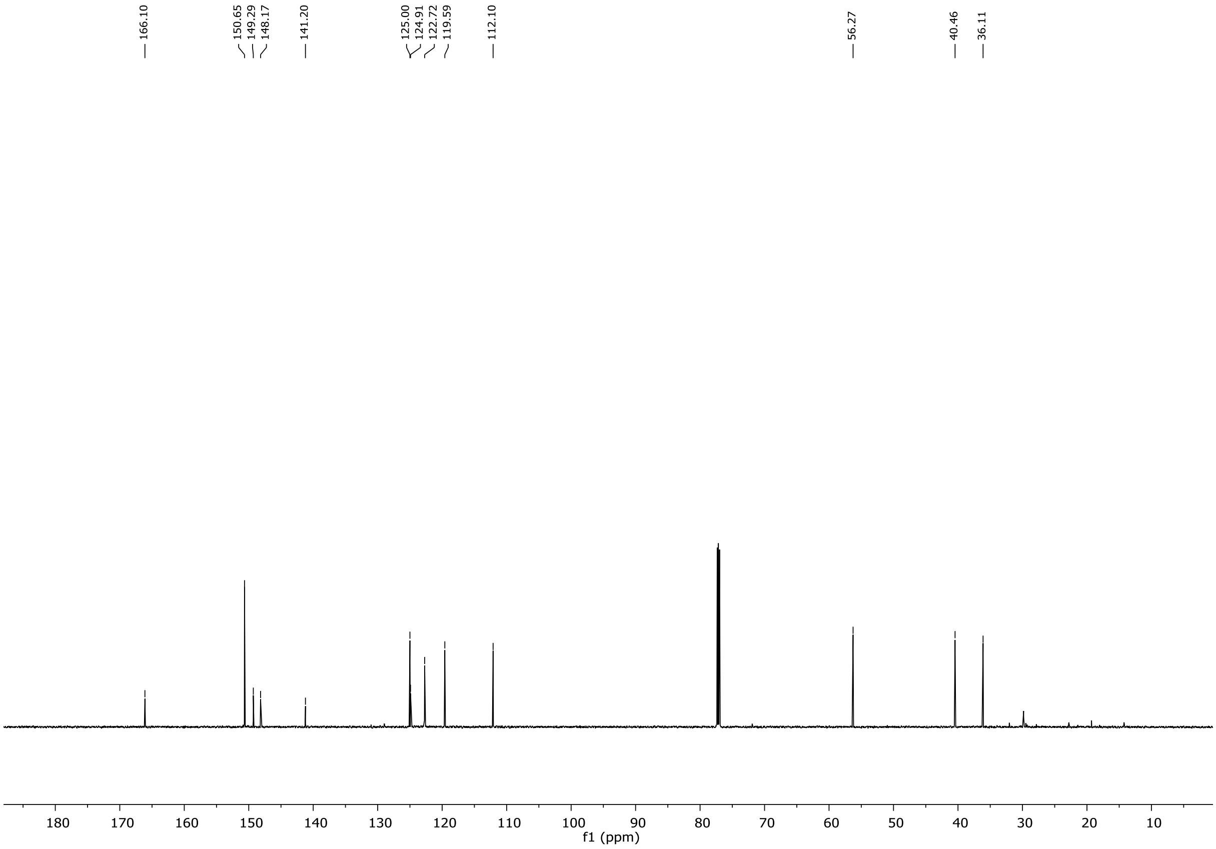

6-*tert*-Butyl-4-(pyridin-4-yl)chroman-2-one 3af

^{1}H NMR (CDCl$_3$, 700 MHz)

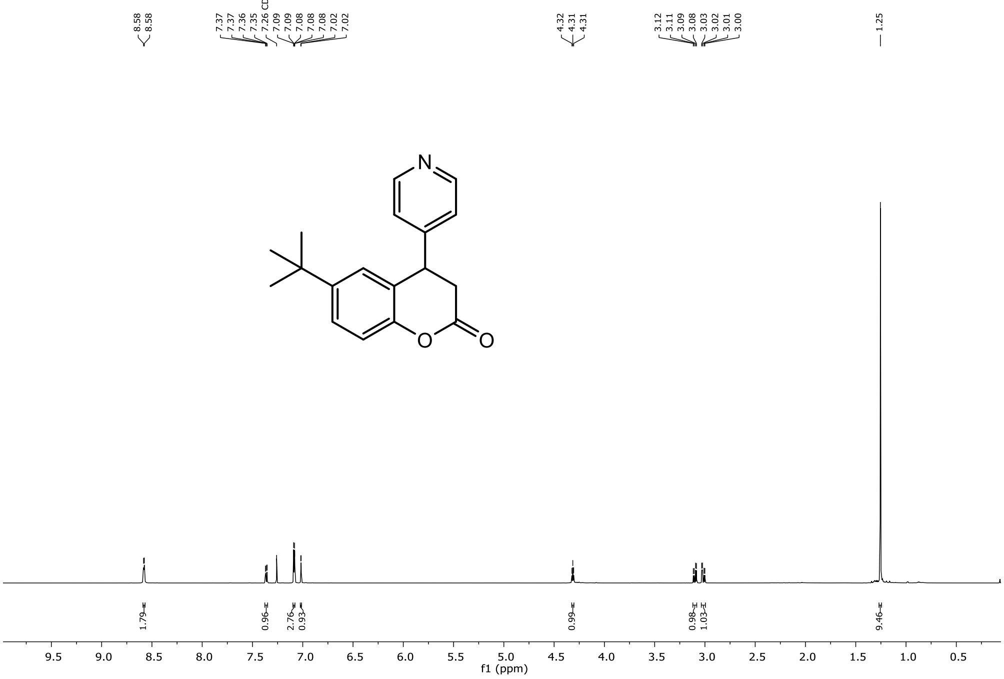

^{13}C {^{1}H} NMR (CDCl$_3$, 176 MHz)

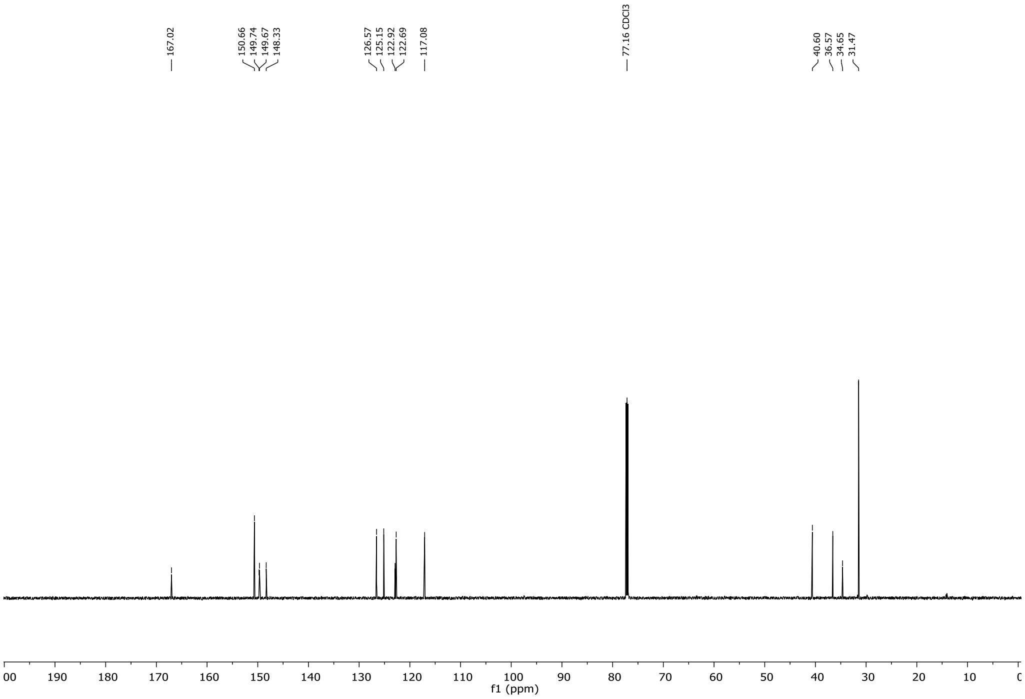

6-Fluoro-4-(pyridin-4-yl)chroman-2-one 3ag

^{1}H NMR ($CDCl_3$, 700 MHz)

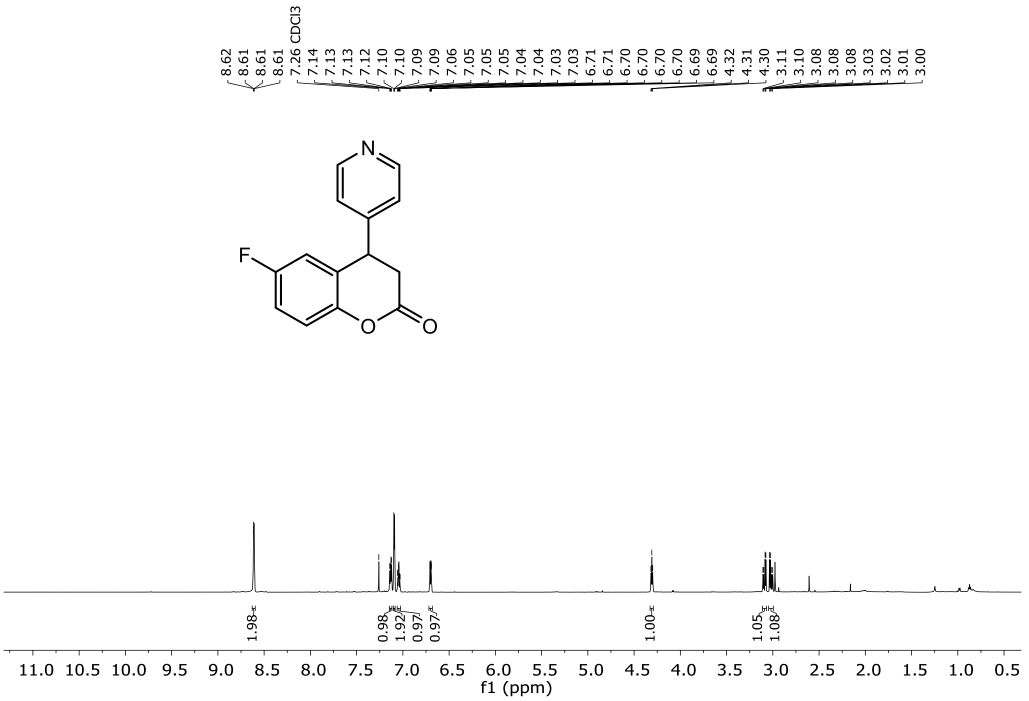

^{13}C {^{1}H} NMR ($CDCl_3$, 176 MHz)

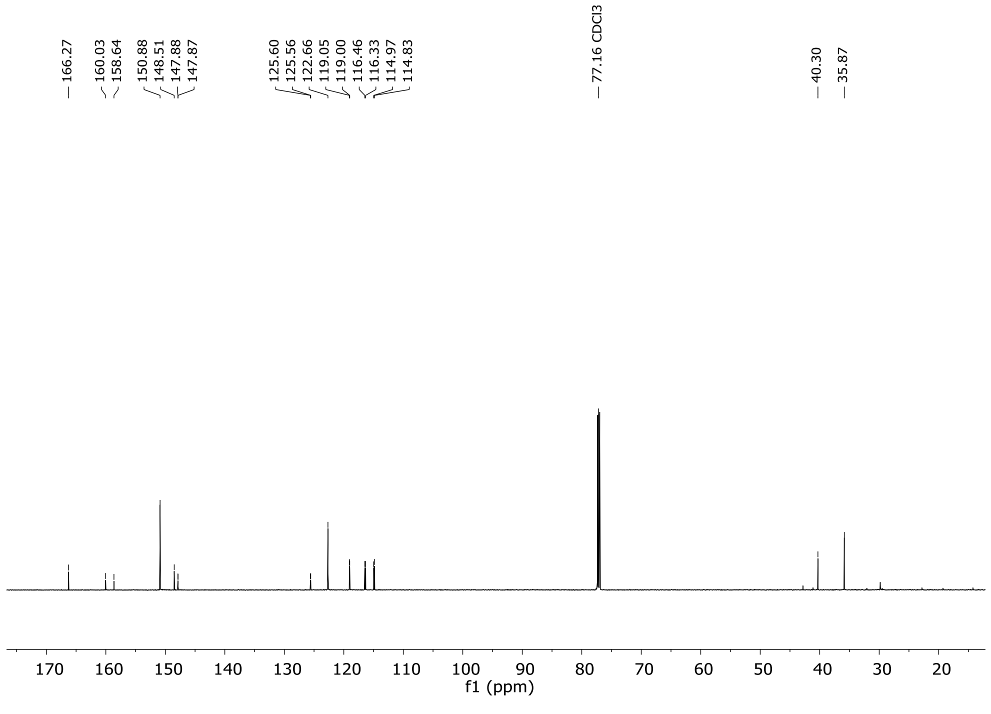

7-Bromo-4-(pyridin-4-yl)chroman-2-one 3ah

^{1}H NMR ($CDCl_3$, 700 MHz)

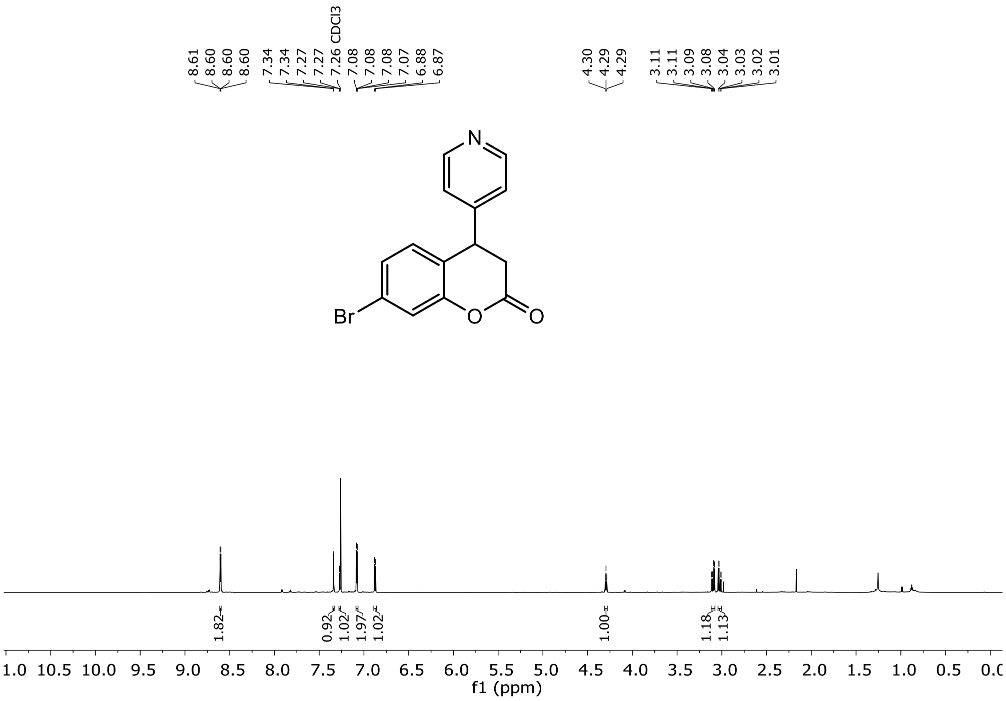

^{13}C {^{1}H} NMR ($CDCl_3$, 176 MHz)

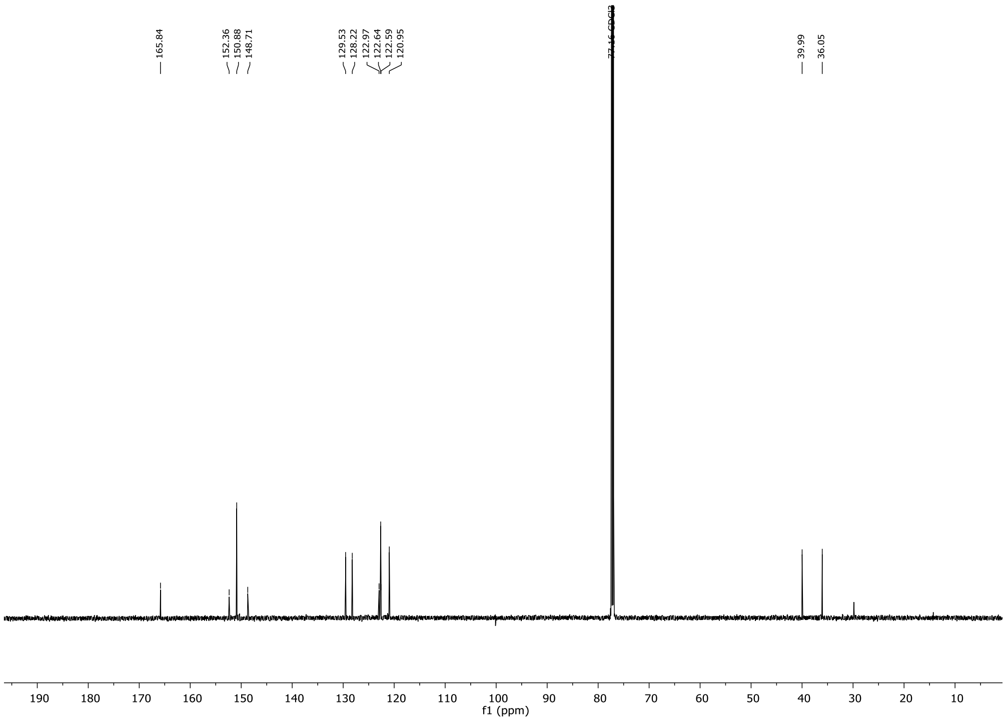

8-Bromo-4-(pyridin-4-yl)chroman-2-one 3ai

^{1}H NMR ($CDCl_3$, 700 MHz)

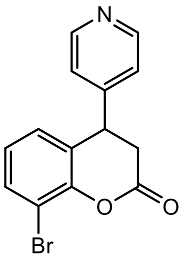

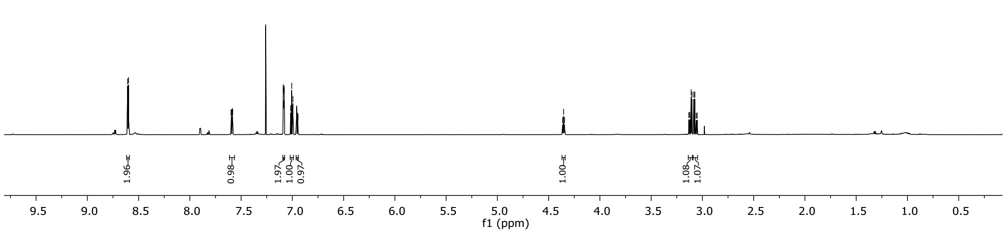

^{13}C {^{1}H} NMR ($CDCl_3$, 176 MHz)

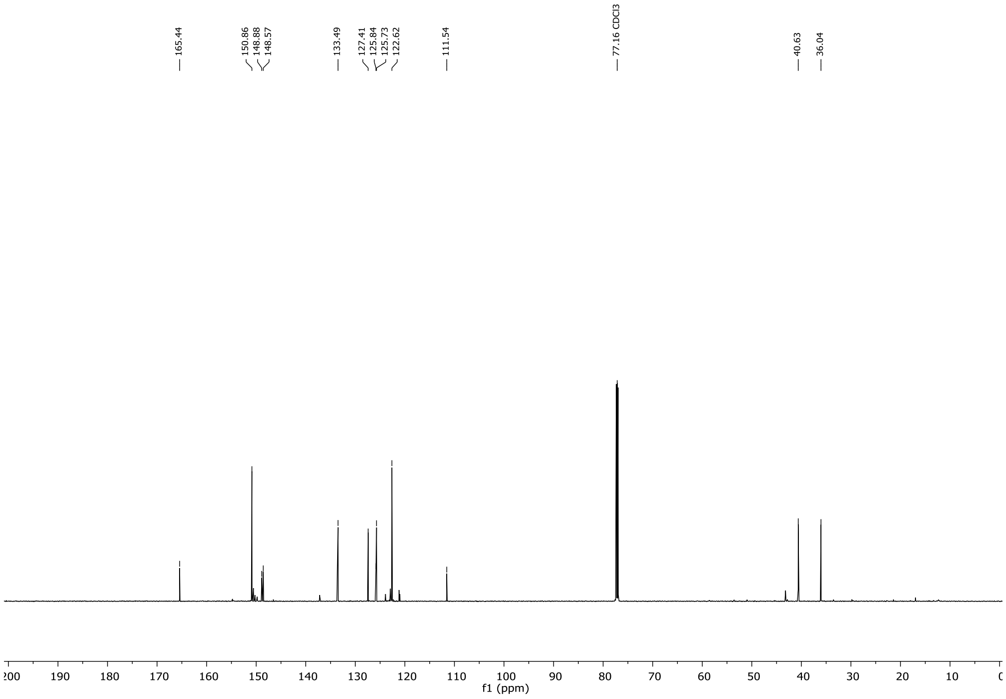

6-Chloro-4-(pyridin-4-yl)chroman-2-one 3aj

^{1}H NMR ($CDCl_3$, 700 MHz)

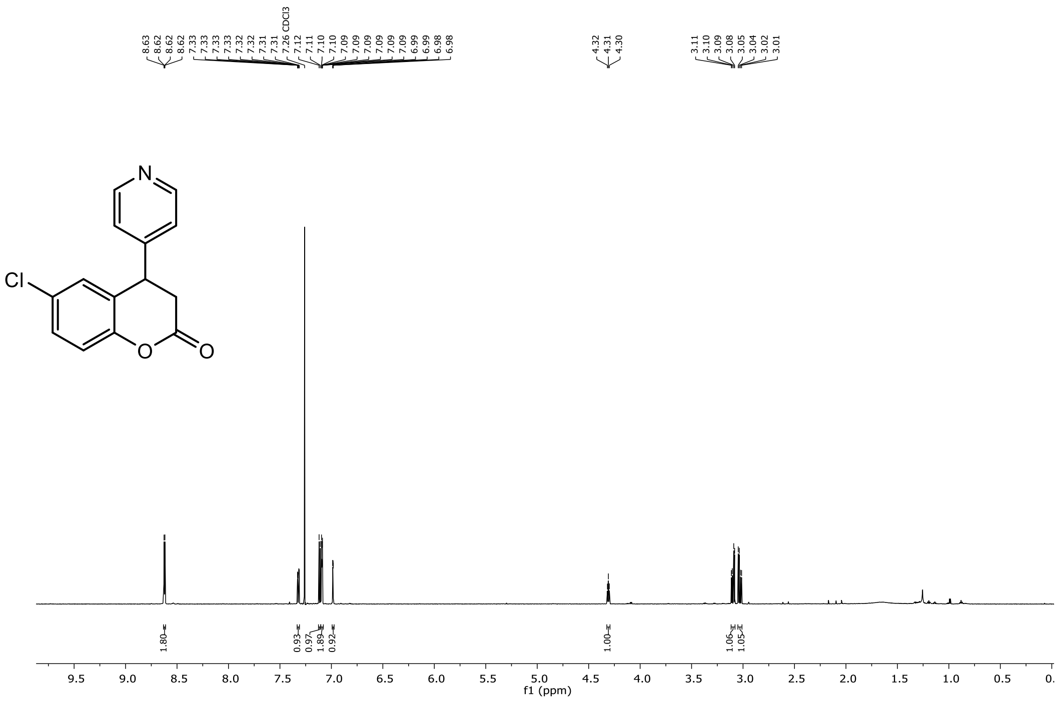

^{13}C {^{1}H} NMR ($CDCl_3$, 176 MHz)

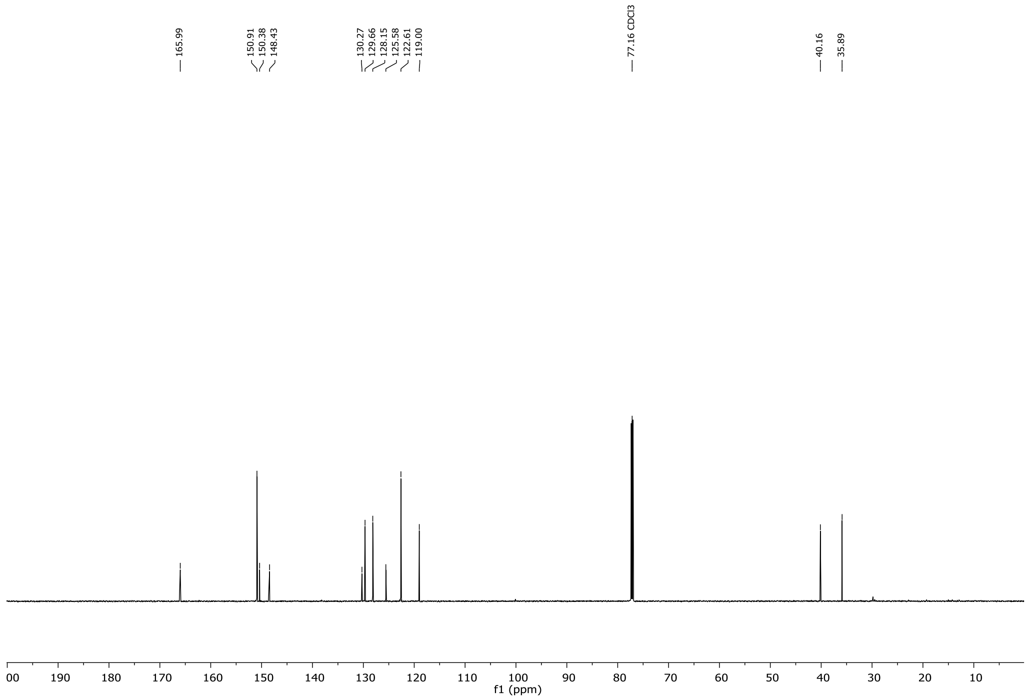

8-Chloro-4-(pyridin-4-yl)chroman-2-one 3ak

^{1}H NMR ($CDCl_3$, 700 MHz)

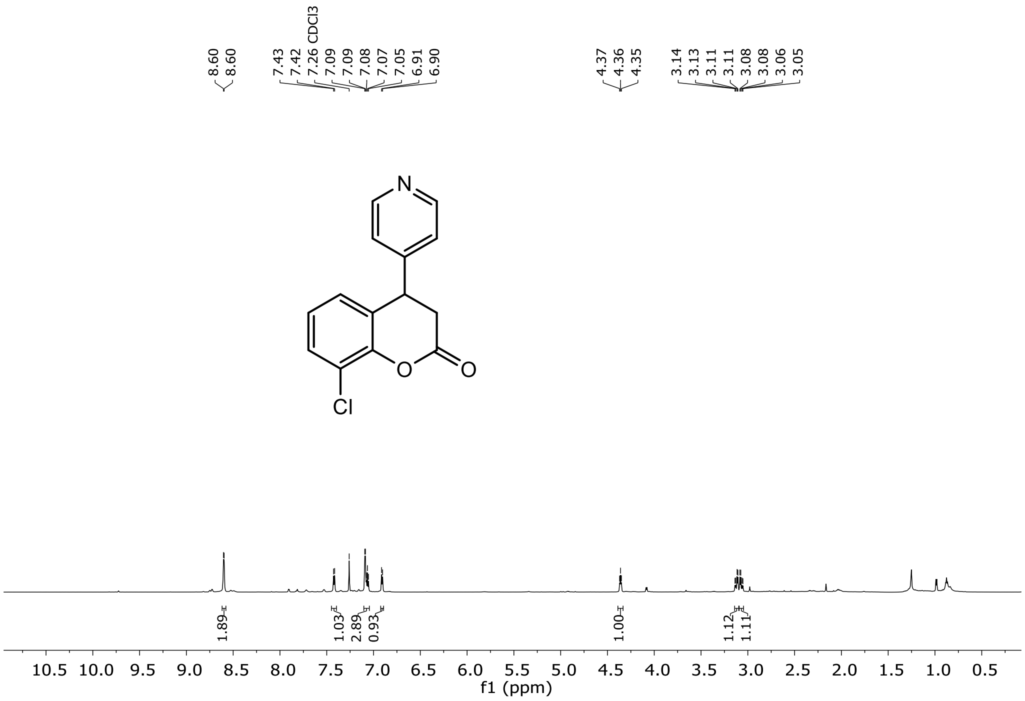

^{13}C {^{1}H} NMR ($CDCl_3$, 176 MHz)

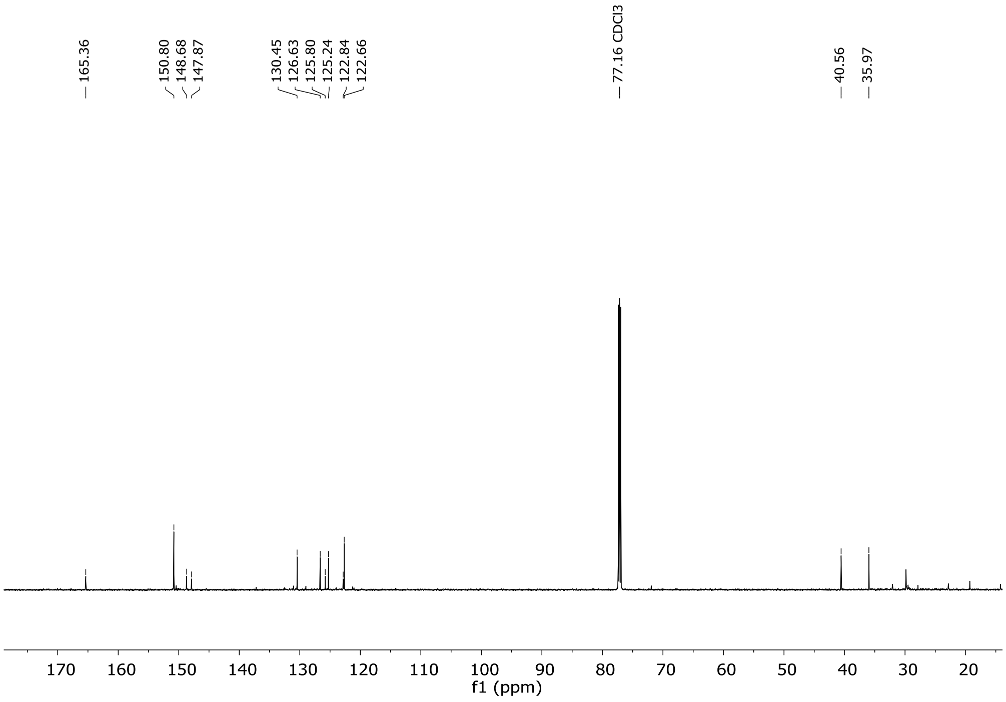

5,7-Methoxy-4-(pyridin-4-yl)chroman-2-one 3am

^{1}H NMR ($CDCl_3$, 700 MHz)

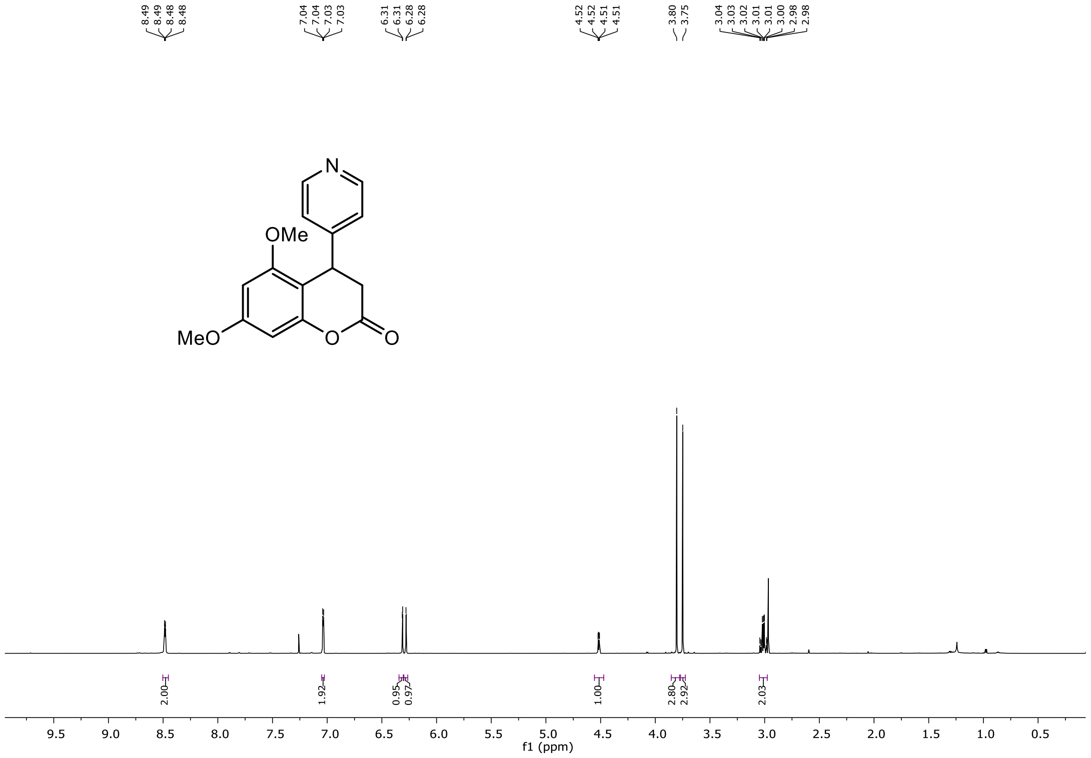

^{13}C {^{1}H} NMR ($CDCl_3$, 176 MHz)

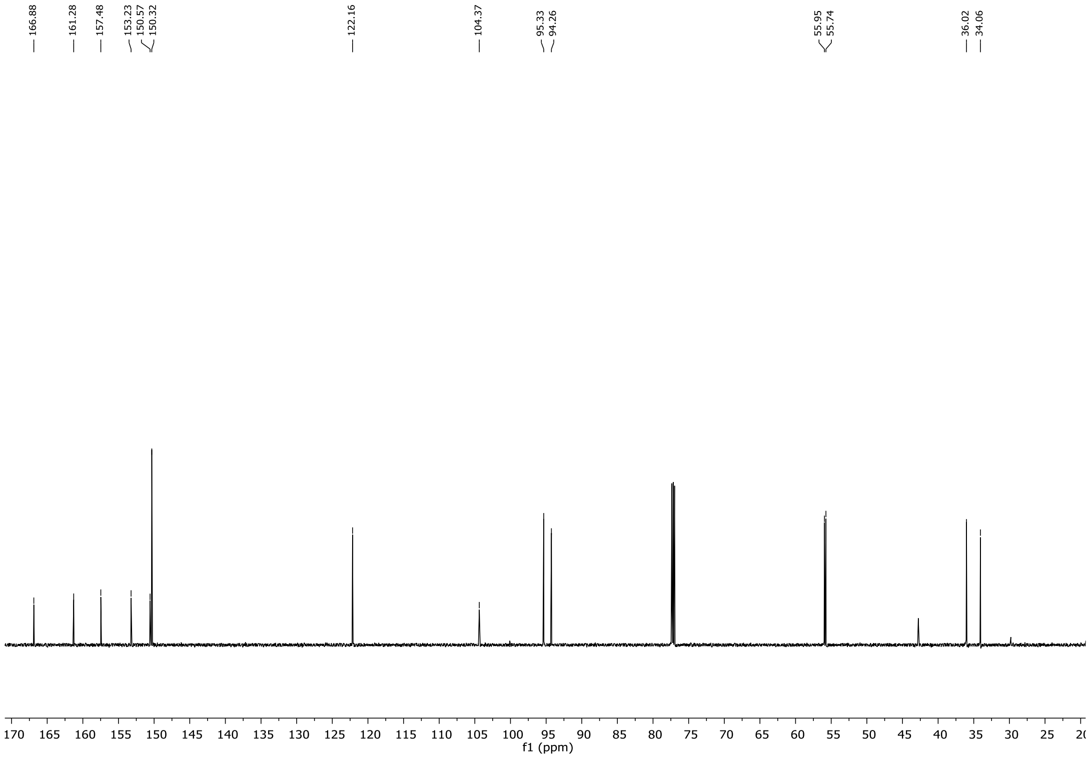

1,2-Dihydro-3*H*-benzo[f]-1-(pyridin-4-yl)chromen-3-one 3al

^{1}H NMR ($CDCl_3$, 700 MHz)

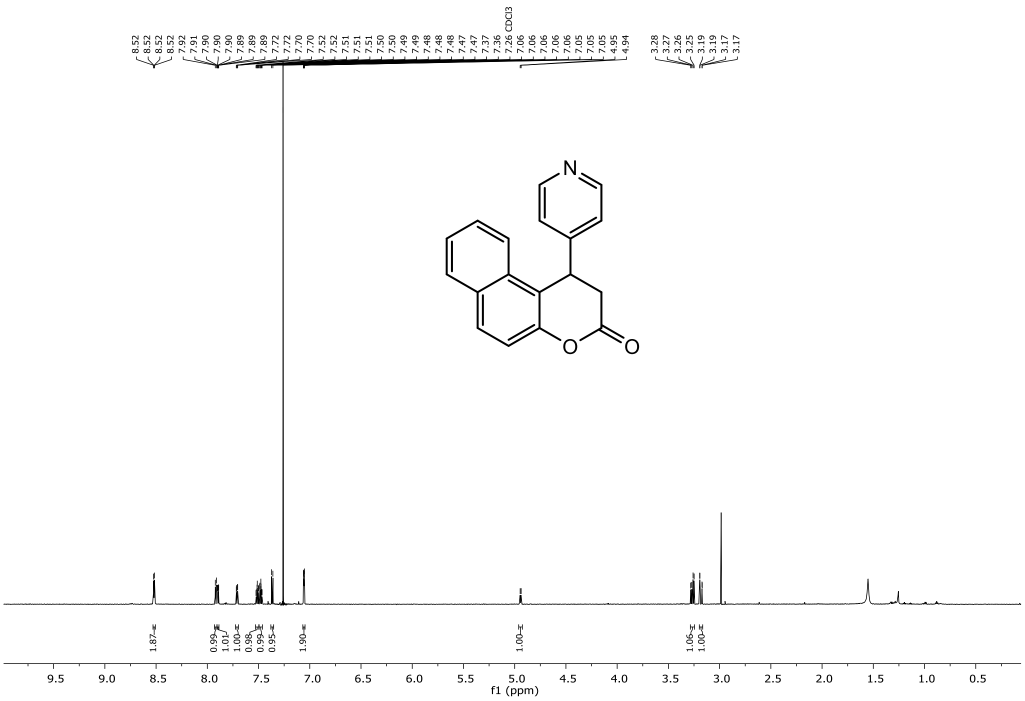

^{13}C {^{1}H} NMR ($CDCl_3$, 176 MHz)

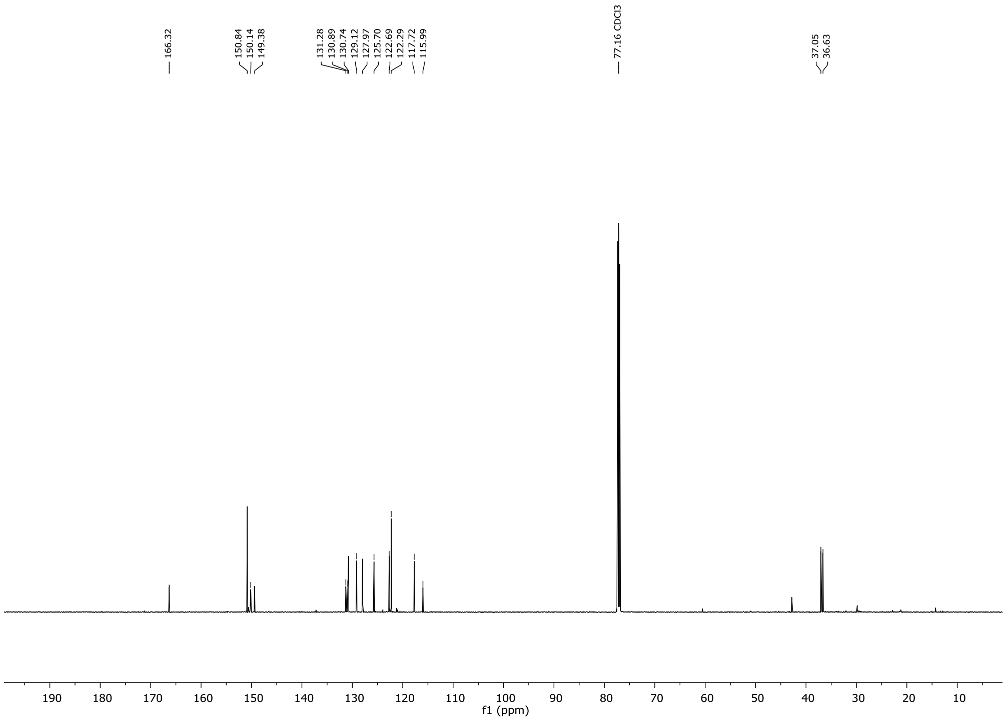

4-(Pyridin-2-yl)chroman-2-one 3ba

^{1}H NMR ($CDCl_3$, 700 MHz)

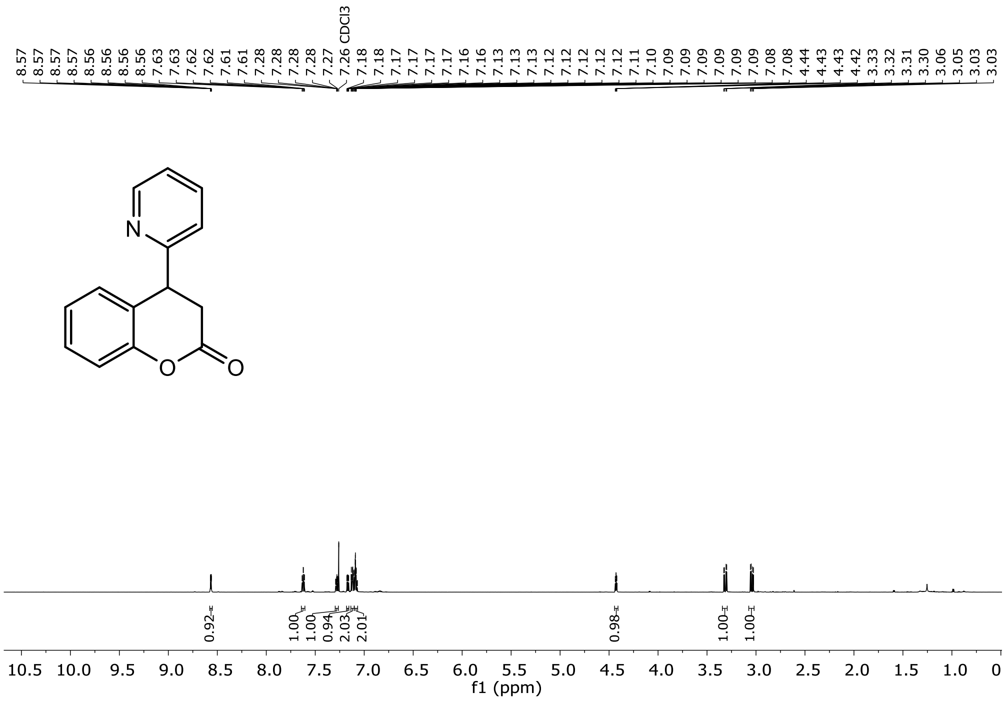

^{13}C {^{1}H} NMR ($CDCl_3$, 176 MHz)

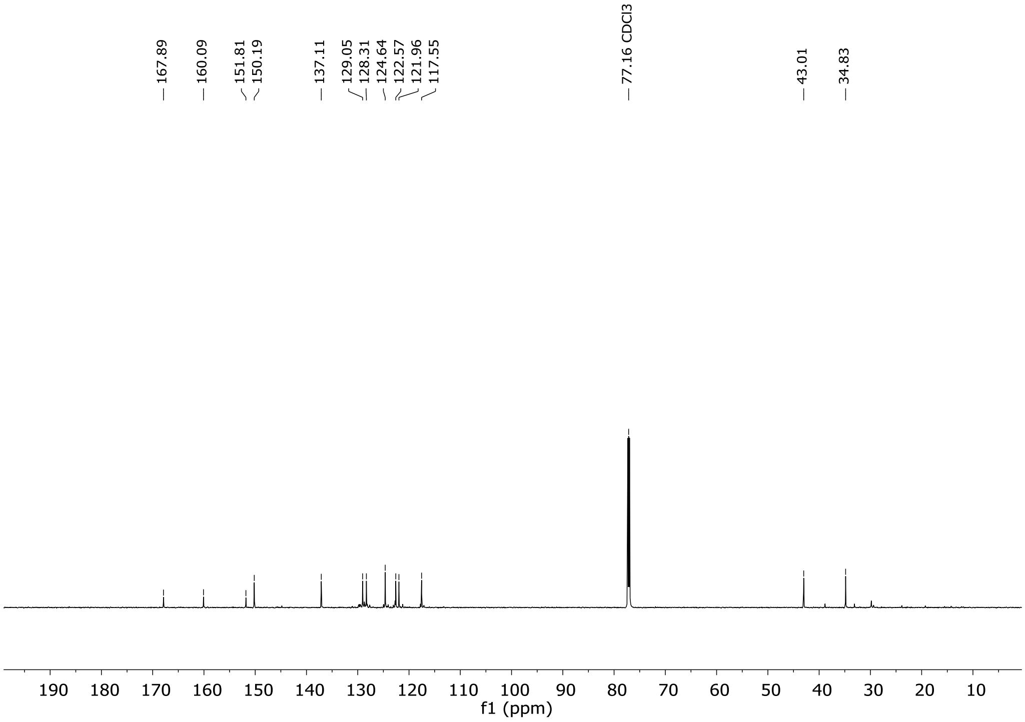

4-(Pyrimidin-2-yl)chroman-2-one 3ca

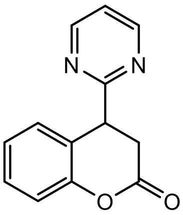

^{1}H NMR ($CDCl_3$, 700 MHz)

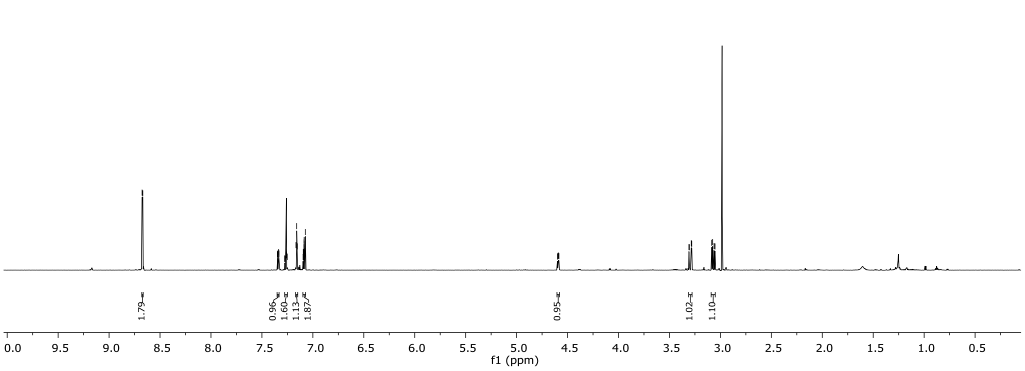

^{13}C {^{1}H} NMR ($CDCl_3$, 176 MHz)

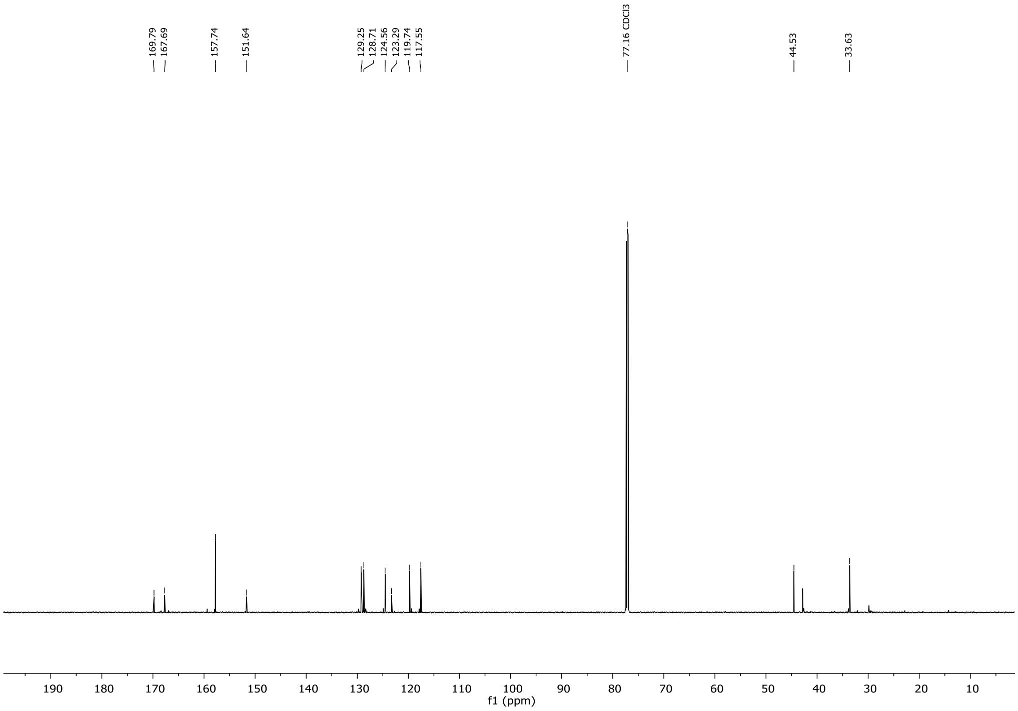

Supplement: Supplementary file 1 — jo2c00683_si_001.pdf [file jo2c00683_si_001.pdf]
